# Advances in the Tribological Performance of Graphene Oxide and Its Composites

**DOI:** 10.3390/ma18153587

**Published:** 2025-07-30

**Authors:** Mayur B. Wakchaure, Pradeep L. Menezes

**Affiliations:** Department of Mechanical Engineering, University of Nevada, Reno, NV 89557, USA

**Keywords:** graphene oxide (GO), GO-based composites, functionalized graphene oxide, self-lubricating materials

## Abstract

Graphene oxide (GO), a derivative of graphene, has attracted significant attention in tribological applications due to its unique structural, mechanical, and chemical properties. This review highlights the influence of GO and its composites on friction and wear performance across various engineering systems. The paper explores GO’s key properties, such as its high surface area, layered morphology, and abundant functional groups. These features contribute to reduced shear resistance, tribofilm formation, and improved load-bearing capacity. A detailed analysis of GO-based composites, including polymer, metal, and ceramic matrices, reveals those small additions of GO (typically 0.1–2 wt%) result in substantial reductions in coefficient of friction and wear rate, with improvements ranging between 30–70%, depending on the application. The tribological mechanisms, including self-lubrication, dispersion, thermal stability, and interface interactions, are discussed to provide insights into performance enhancement. Furthermore, the effects of electrochemical environment, functional group modifications, and external loading conditions on GO’s tribological behavior are examined. Despite these advantages, challenges such as scalability, agglomeration, and material compatibility persist. Overall, the paper demonstrates that GO is a promising additive for advanced tribological systems, while also identifying key limitations and future research directions.

## 1. Introduction

Tribology is the study of friction, wear, and lubrication [1], three fundamental phenomena that influence the performance and durability of mechanical systems. Tribology plays a vital role in various industries such as automotive [2], aerospace [3], biomedical [4], and manufacturing [5]. Mechanical systems’ efficiency majorly depends on tribological aspects. Therefore, studying tribology and its effect on various materials is important to enhance performance and reduce energy losses [6,7,8,9,10,11].

Graphene oxide (GO) is one of the materials that has garnered significant interest due to its unique structural, chemical, and mechanical characteristics [12,13]. GO is a two-dimensional material derived from graphene [14]. Its oxygen-containing functional groups have excellent dispersibility, tunable surface chemistry, and improved mechanical properties. Comparing GO with pure graphene, GO is hydrophilic, which allows it to interact effectively with various lubricants, base oils, and polymer matrices [15]. GO has the ability to change its surface chemistry [16]. This makes it adaptable and able to be used for specific desired industrial applications in different operating environments.

GO can reduce friction and wear, which can lead to increased efficiency and durability [17]. Commonly used lubricants have limitations such as thermal degradation, low dispersion, and limitations with bearing capacity. In tribology, GO is widely used to increase efficiency and durability in mechanical components [18]. When compared to other lubricants, GO provides good lubrication, thermal stability, and greater load-bearing capacity [19]. Using lubricants or coatings over GO creates a protective film over its surface, which minimizes direct contact with metal, thus decreasing component wear and tear and increasing their lifespan [20].

GO possesses properties such as high mechanical strength, a high Young’s modulus, and notable flexibility. These mechanical characteristics make it suitable for use as a solid lubricant and as a reinforcing agent in composite materials [21,22]. Unlike solid lubricants such as graphite or molybdenum disulfide, GO maintains stability under extreme weather conditions, such as high temperature or pressure, and in chemically reactive environments [23]. This special feature of GO to maintain its stability can be used in various applications such as space technology, high-speed machining, and biomedical implants. We can also add GO to common lubricants as an additive to improve their tribological performance [18,19].

GO has a high surface area and layered structure, so it can reduce shear resistance in between contact surfaces and decrease the friction coefficient [15]. Additionally, GO can form tribofilms on sliding interfaces, enhancing wear resistance, preventing surface degradation, and improving efficiency in energy-intensive applications.

GO-based coating also has great potential in tribology. By adding GO in the coatings, scientists have developed highly advanced wear-resistant surfaces, which provide long-term protection from abrasive and adhesive wear [24]. These GO-based coatings not only have good mechanical durability but also provide significant resistance to corrosion, oxidation, and high-temperature degradation [25]. This feature of GO as a self-lubricating material in coatings has potential in a wide range of industrial applications, particularly in automotive engines, cutting tools, and biomedical devices [26].

The advantages of GO include its capacity for operationalization. The surface chemistry of GO can be customized to improve its compatibility with the different base materials, lubricants, and composite materials. Scientists have upgraded GO’s adhesion, dispersion, and stability, thus optimizing its tribological properties [27]. This adaptive nature of GO makes it ideal for usage with hybrid composites, where it can be blended with polymers, metals, and ceramics to succeed in high-performance tribological materials with good durability and wear resistance.

Despite these optimistic properties, the widespread usage of GO is limited in tribology. One vital limitation is the stability of the GO during operational conditions. Overlong exposure to high temperatures, humidity, and oxidative environments may lead to degradation of GO, which affects its performance as a lubricant or coating material [28]. Another major concern is the cost and scalability of GO production. Although various synthesis methods are available, producing high-quality GO at an industrial scale while maintaining cost-effectiveness remains a significant challenge that needs to be addressed [29].

This analysis focuses on providing a comprehensive overview of the approach to the tribological performance of GO and its composites. The objectives include exploring fundamental properties of GO relevant to tribology, analyzing its application across different lubrication systems, and discussing the mechanisms that contribute to its tribological effectiveness. Furthermore, this analysis highlights challenges, limitations, and future supervision for GO-based tribological materials, paving the way for further research and its adoption in industry. Moreover, GO continues to evolve; its role in tribology is expanding, offering sustainable and cost-effective alternatives to regularly used lubricants and wear-resistant materials. The growth of novel GO-based composites, advancements in the synthetic techniques, and exploration of their application in the industries will drive further progress in this field. By using the unique properties of GO, researchers and engineers can develop future-generation tribological materials that amplify efficiency, reduce energy losses, and increase the reliability of mechanical systems.

## 2. Overview of Production Methods of GO-Based Materials and Composites

GO-based materials, composites, and lubricants are prepared through diverse methods. GO synthesis primarily employs chemical oxidation techniques such as the Hummers method or modified Hummers methods, using graphite as a precursor, oxidized by strong acids (e.g., H_2_SO_4_) and oxidants (KMnO_4_). These processes introduce functional groups (epoxy, hydroxyl, and carboxyl) that significantly influence GO’s dispersibility and reactivity [12,13,14,15,21,22,23,28].

Lubricants containing GO typically use ultrasonication methods to disperse GO sheets uniformly into base lubricants, enhancing stability and preventing aggregation. Surfactants or stabilizers can also be used to achieve stable dispersions suitable for tribological applications [18,19,24].

GO-based polymer composites are commonly fabricated via solution blending, melt compounding, or in situ polymerization. Solution blending involves mixing GO dispersed in a solvent with polymer solutions, followed by casting or evaporation to create composite films [22].

Metal and ceramic matrix composites reinforced with GO are typically produced using powder metallurgy, vacuum infiltration, chemical vapor deposition (CVD), or spark plasma sintering (SPS). Powder metallurgy involves mixing GO with metal or ceramic powders, then compacting and sintering under controlled conditions. Vacuum infiltration techniques impregnate porous preforms with GO dispersions, enhancing the composite’s structural properties and tribological performance [22,26].

GO-based coatings are fabricated using layer-by-layer assembly, spray coating, electrophoretic deposition, or electrochemical deposition. These methods enable uniform application and robust adhesion of GO to various substrates, suitable for diverse industrial coatings [24,25,26,27,28,29].

## 3. Significance of Graphene Oxide in Tribology

GO has emerged as a highly promising material in tribology due to its exceptional structural, chemical, and mechanical properties. Tribology plays a critical role in enhancing the efficiency and longevity of mechanical components [21]. The incorporation of GO in tribological applications has gained significant attention as it offers superior lubrication, wear resistance, and friction reduction in various systems.

**Figure 1:**
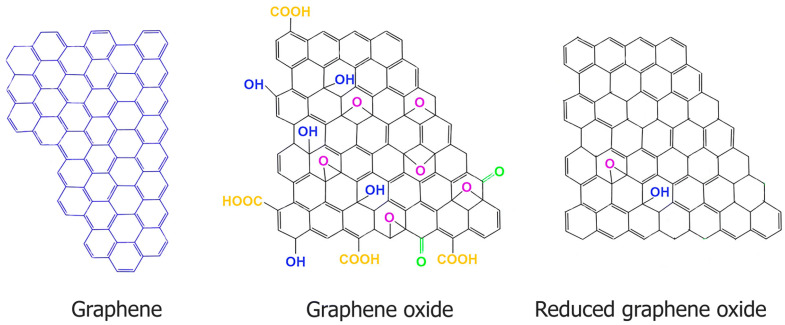
Chemical structure of graphene, graphene oxide (GO), and reduced graphene oxide (rGO). Reproduced from [30] under the terms of the Creative Commons Attribution License (CC BY).

GO possesses a unique two-dimensional (2D) layered structure with a high surface area, which allows it to form protective films on contact surfaces, thereby minimizing direct metal-to-metal contact and reducing wear [24]. Additionally, the presence of oxygen-containing functional groups on GO enhances its dispersibility in both aqueous and non-aqueous media, making it an ideal additive in lubricants and coatings [28]. Figure 1 shows the chemical structure of graphene, graphene oxide (GO), and reduced graphene oxide (rGO). Graphene is a single layer of carbon atoms with a perfect sp^2^ structure, offering excellent conductivity and strength but poor dispersibility in water. GO contains oxygen functional groups such as hydroxyl and carboxyl, making it hydrophilic and chemically reactive but less conductive. Reduced graphene oxide is partially restored GO, with improved conductivity and fewer oxygen groups, though not as pristine as graphene. These structural differences influence their applications in electronics, composites, and lubrication.

The superior mechanical properties of GO, including its high Young’s modulus and excellent flexibility, contribute to its effectiveness as a solid lubricant and reinforcement in composites. GO-based lubricants and coatings have demonstrated significant reductions in the coefficient of friction and wear rate when compared to conventional materials [31]. Furthermore, the thermal stability and thermal conductivity of GO aid in dissipating heat generated during frictional interactions, preventing localized overheating and material degradation [32]. Another critical advantage of GO in tribology is its potential for functionalization. By modifying its surface chemistry, GO can be tailored to enhance its tribological performance in specific environments, such as high-temperature, high-pressure, or chemically reactive conditions. This adaptability makes GO an excellent candidate for advanced lubricant formulations and tribological applications.

Reduced graphene oxide is obtained by chemically or thermally reducing GO, which restores portions of its sp^2^ carbon network and enhances its thermal and electrical conductivity compared to unreduced GO [14,18]. This structural change also improves its mechanical stiffness and load-bearing capacity, making it suitable for high-load tribological applications [23,24]. However, the reduction process decreases the number of oxygen-containing groups, reducing hydrophilicity and making rGO less dispersible in polar media [19,22]. Despite this, rGO demonstrates lower coefficients of friction (as low as 0.05) and more stable tribofilm formation under dry or boundary lubrication regimes [25,26]. Due to its tendency to agglomerate, dispersion techniques such as surfactant treatment or polymer grafting are often employed [27]. In comparison, GO is more favorable in applications requiring high dispersion stability and interfacial reactivity, while rGO is advantageous where enhanced mechanical strength and thermal endurance are prioritized [28,29].

Overall, the integration of GO in tribology represents a significant advancement in developing high-performance, energy-efficient, and durable lubrication solutions. As research continues to explore novel GO-based composites and functionalization techniques, its role in tribological applications is expected to expand further, offering sustainable and cost-effective alternatives to traditional lubricants and wear-resistant materials.

## 4. Properties of Graphene Oxide Relevant to Tribology

**Figure 2:**
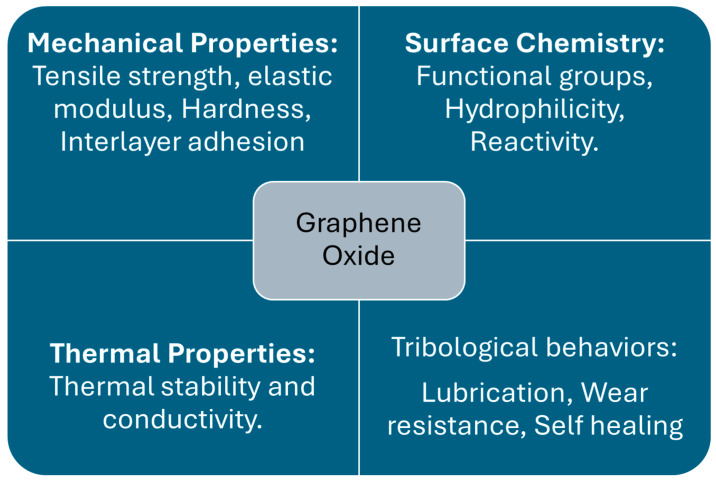
Overview of important properties of graphene oxide.

GO is the ideal material because of its unique combination of properties. It has a large surface area, and it contains oxygen as a functional group [33]. This increases interaction with other materials, which leads to improved lubrication and reduced friction. For wear resistance, GO’s mechanical strength and toughness make it an attractive material [34], whereas its self-lubricating properties help minimize friction in contact surfaces [35]. GO has good thermal stability, allowing it to withstand high temperature conditions. It is also hydrophilic in nature, which makes it effective in aqueous lubrication systems. These features make GO an ideal candidate for enhancing the tribological performance of materials. Table 1 lists the physical properties of graphene oxide and its approximate values based on research. Figure 2 indicates the overview of GO properties important for its performance, which are detailed further in Section 3.

### 4.1. Structural and Chemical Properties

GO consists of single-layer carbon sheets in a hexagonal lattice, similar to graphene, but decorated with oxygen-containing groups such as epoxides, hydroxyls, and carboxyls [42]. These groups introduce defects and increase interlayer spacing, making GO more hydrophilic, chemically reactive, and easily dispersible in polar solvents [41]. By controlling oxidation or applying reduction, GO’s mechanical and electrical properties can be tuned while maintaining strong interfacial bonding with polymers, metals, and ceramics. These structural and chemical features make GO highly versatile for tribological and composite applications. Table 2 and Table 3summarize its key structural and chemical characteristics.

### 4.2. Mechanical Properties

GO’s mechanical properties depend on its oxidation level and defects from functional groups. Although its tensile strength and stiffness are lower than pristine graphene, GO maintains enough flexibility and toughness for composites, with a Young’s modulus of 200–250 GPa and tensile strength of 100–200 MPa [51,52,53,54]. In polymer or metal matrices, GO enhances load transfer, crack deflection, and stress distribution, making it effective for reinforcing coatings and tribological materials under moderate stresses [54,55,56]. Table 4 shows the mechanical properties of GO. Pristine graphene has far higher tensile strength (~130 GPa) than GO, which is weakened by oxidation defects but is still strong enough for composite reinforcement. Although more brittle, GO retains flexibility and interlayer adhesion, making it useful in coatings and layered systems. Its compressive strength varies by form—GO aerogels, for example, are highly resilient. Reduction or chemical modification allows tuning GO’s mechanical properties for tribological and polymer applications. Table 4 summarizes the typical mechanical properties of GO, including tensile strength, elastic modulus, and compressive resilience reported in the literature.

### 4.3. Thermal Stability and Conductivity

GO exhibits moderate thermal stability, significantly lower than pristine graphene due to its oxygen-containing functional groups (epoxy, hydroxyl, carboxyl), which start decomposing around 150–200 °C and are largely reduced by 300–400 °C, depending on environment and heating rate [39,58,59,60]. This decomposition releases CO, CO_2_, and H_2_O, partially restores the sp^2^ network, and transforms GO into rGO with improved thermal conductivity and stability [39,59,60,61,62]. At higher temperatures (600–800 °C), GO or rGO can react with carbide-forming elements such as Ti, Cr, Mo, and W to form hard carbides (e.g., TiC, Cr_3_C_2_, Mo_2_C, WC) that enhance wear resistance in composites [21,22,23]. However, excessive carbon exposure or prolonged heat can destabilize these carbides, creating brittle carbon-rich interfaces and compromising mechanical integrity [25,26,27]. Additionally, GO is an electrical insulator due to disrupted π-conjugation, but reduction significantly improves its conductivity, making it suitable for energy storage and flexible electronics [60,61]. Understanding and controlling these thermal and chemical behaviors is critical for designing GO-based composites for high-temperature tribological applications. Table 5 summarizes GO’s thermal properties.

### 4.4. Surface Chemistry and Functionalization

GO contains oxygen functional groups such as epoxides, hydroxyls, and carboxyls, which significantly alter its surface chemistry [16]. These groups increase GO’s hydrophilicity and enable strong hydrogen bonding, affecting its stability in aqueous and polar solvents [41]. The negative surface charge, primarily due to deprotonated carboxyl groups, enhances electrostatic interactions, making GO highly tunable for various applications.

GO’s functional groups allow for covalent functionalization, where chemical reactions with amines, thiols, or silanes modify its properties for enhanced mechanical strength, lubrication, and compatibility with polymer matrices. Alternatively, non-covalent interactions, such as π-π stacking and van der Waals forces, enable integration with organic and inorganic materials without altering their core structure [64,65]. This is particularly useful in tribological applications, where maintaining structural integrity is crucial for friction and wear performance.

By adjusting functionalization methods, GO can be tailored for specific applications, including self-lubricating coatings [66] and reinforced composites [67]. Reduction processes can partially restore its sp^2^ carbon network, improving conductivity while maintaining essential surface interactions [59]. These chemical modifications are key to optimizing GO’s role in advanced tribological systems. While functionalization of GO enhances its compatibility with polymer matrices, metal ions, and lubricants, it also introduces several trade-offs. Covalent functionalization often disrupts the sp^2^ carbon lattice, leading to a loss in electrical conductivity and mechanical strength. In non-covalent approaches, π–π stacking or hydrogen bonding can cause restacking or aggregation of GO sheets, especially under high-temperature or shear conditions, reducing effective surface area. Moreover, excessive surface modification can hinder the tribofilm-forming ability of GO or alter its lubricating behavior unpredictably. These effects necessitate a balance between functional group density and retention of intrinsic 2D characteristics, which remains a key design challenge in GO-based tribological systems [26,27,28,29].

## 5. Tribological Applications of Graphene Oxide

GO has gained significant attention in tribology due to its unique physicochemical properties, including its high mechanical strength, tunable surface chemistry, and excellent lubricating performance. Its applications span multiple tribological domains, ranging from solid lubricants to self-lubricating composites and coatings. Table 6 gives an overview of the GO application area in tribology, including its benefits and applications. This section discusses the major tribological applications of GO in detail.

### 5.1. Solid Lubricant Properties

GO exhibits remarkable solid lubricant properties due to its lamellar structure, low shear strength, and ability to form protective films on contact surfaces [21]. The oxygen-containing functional groups in GO facilitate strong adhesion to metal, ceramic, and polymer surfaces, reducing direct asperity contact and minimizing wear [72]. Studies have shown that GO can significantly reduce friction and wear under dry conditions, making it a promising candidate for dry lubrication in high-precision mechanical systems [73]. Compared to conventional solid lubricants such as molybdenum disulfide (MoS_2_) and graphite, GO offers superior oxidation resistance and tunable surface interactions, enhancing its tribological performance in diverse applications [74].

**Figure 3:**
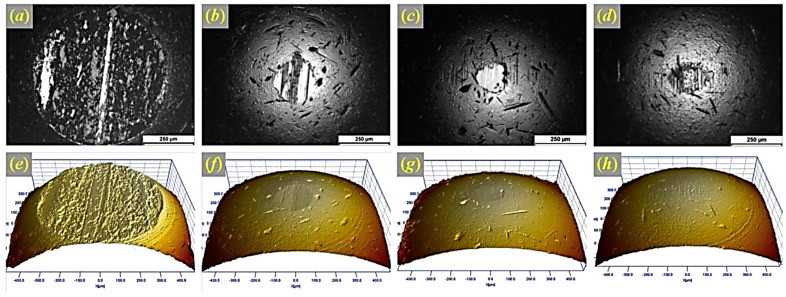
Optical micrographs and 3D surface reconstructions showing how composition affects wear scars on stationary specimens. (**a**,**e**) Unlubricated sliding results in a deep, rough scar. (**b**,**f**) Graphene lubrication reduces scar size and smooths the surface. (**c**,**g**) Adding PVDF further minimizes the scar. (**d**,**h**) The composite coating nearly eliminates visible wear, preserving the surface integrity. Reproduced from [77] with permission from Elsevier, © 2017. All rights reserved.

Additionally, GO exhibits self-healing behavior in tribological contacts, where the exfoliated nanosheets replenish worn-out regions, thereby extending service life [75]. The high surface energy of GO enables it to form stable lubricating layers under varying load conditions [76]. As shown in Figure 3, optical micrographs and 3D surface reconstructions illustrate how composition significantly influences wear scar formation under dry sliding. The unlubricated specimen exhibits a deep, rough wear scar, whereas the use of graphene and PVDF notably reduces wear depth and roughness. Incorporating a composite coating further minimizes damage, effectively preserving the surface integrity. These observations demonstrate the superior wear resistance imparted by graphene-based lubricants and coatings [77].

Moreover, recent advancements in GO functionalization have improved its dispersibility and compatibility with different substrates, further broadening its applicability as a solid lubricant in engineering systems. Table 7 highlights material pairs where graphene oxide has been applied as a solid lubricant, showing significant reductions in friction and wear, particularly in dry contact conditions.

To better understand the effectiveness of GO as a solid lubricant, it is important to compare its performance with other widely used solid lubricants such as molybdenum disulfide (MoS_2_), graphite, hexagonal boron nitride (h-BN), and PTFE. Table 8 summarizes this comparison based on key tribological and environmental properties, using data reported in the literature.

As shown in Table 8, GO demonstrates competitive friction and wear reduction performance, with additional benefits such as self-healing behavior, surface functionalization capability, and higher stability in humid and oxidative environments. These characteristics make GO a promising alternative to conventional solid lubricants, especially in conditions where traditional materials such as MoS_2_ are prone to degradation.

### 5.2. Additives in Lubricants

GO is widely investigated as an additive in lubricating oils and greases due to its ability to improve tribological performance. When dispersed in base oils, GO sheets create a protective tribofilm that enhances load-bearing capacity and minimizes direct surface contact. Additionally, the functional groups of GO enable stable dispersion in both water-based and oil-based lubricants, preventing agglomeration and ensuring long-term performance [79]. Experimental studies have demonstrated that GO-based lubricants can significantly reduce the coefficient of friction (COF) and wear rate, outperforming traditional additives such as zinc dialkyldithiophosphates (ZDDPs) [80,81]. The synergistic effect of GO with other nanomaterials, such as molybdenum disulfide and carbon nanotubes, further enhances the lubricating efficiency, making it an attractive option for industrial lubrication applications [31,82]. Furthermore, GO enhances the thermal stability and anti-wear properties of lubricants, making them suitable for high-temperature applications. Its ability to interact with metal surfaces at the nanoscale contributes to forming durable tribofilms that reduce adhesion wear [83]. Ongoing research focuses on optimizing GO dispersion techniques and modifying its surface chemistry to improve its solubility and performance in various lubricant formulations.

In addition to pure GO, hybrid nanostructures, such as GO–MoS_2_, GO–carbon nanotubes (CNTs), GO–boron nitride (BN), and GO–SiC, have demonstrated enhanced tribological performance when used as lubricant additives. These hybrids combine the interlayer shear characteristics of GO with the complementary properties of other nanomaterials. For instance, MoS_2_–GO hybrids integrate GO’s oxidative stability with MoS_2_’s ultralow shear strength, offering superior performance under varying environmental conditions, especially where conventional MoS_2_ suffers degradation in humid or oxidative atmospheres [74,80]. The GO–CNT composites provide improved mechanical reinforcement, enhanced dispersion stability, and better thermal conductivity. CNTs act as load-bearing backbones while GO offers film-forming and functionalization advantages, collectively reducing wear and friction under high loads [82]. In GO–BN hybrids, the electrical insulation and chemical inertness of BN complement GO’s triboactive nature, making them suitable for electrically sensitive or corrosive environments. These hybrid systems often exhibit synergistic effects, where the tribological performance surpasses the additive effect of the individual components due to improved tribofilm compactness, interfacial load transfer, and energy dissipation. Additionally, functionalized GO hybrids promote stable dispersion in polar and nonpolar base oils through surface charge tailoring and steric stabilization [79,81], addressing one of the major limitations of nanolubricants: agglomeration over time. As hybrid formulations continue to evolve, their role in next-generation energy-efficient lubrication systems is expected to expand, especially for extreme operating conditions in aerospace, automotive, and high-speed machining applications.

Table 9 presents examples of graphene oxide-based liquid lubricant behavior, showing how small additions of GO can substantially enhance both friction and wear performance.

Recent advancements in the application of GO as a coolant and lubricant additive have shown substantial potential in improving tribological performance across diverse machining and sliding environments. In turning processes, GO nanosheets significantly enhance thermal conductivity and reduce cutting temperatures, ultimately minimizing friction and improving tool life and machining stability, as validated by both experimental and simulation models [87]. Beyond reducing cutting forces, GO-enhanced lubricants have also demonstrated excellent friction-reducing and anti-wear properties, even in challenging environments. In certain cases, superlubricity with extremely low friction has been achieved due to favorable chemical interactions and the formation of protective tribofilms at the interface [88]. This superlubricity can be achieved in solid-liquid systems through the formation of stable tribofilms at the interface, which significantly reduce friction by enabling smooth sliding with minimal shear resistance [89].

Additionally, turning experiments with GO-based nanofluids during Ti-6Al-4V machining demonstrated a reduction in cutting forces and improvement in tool wear resistance [90]. These enhancements were also accompanied by reduced cutting vibrations, better chip control, and smoother cutting conditions. Complementing these findings, graphene-based nanolubricants have shown promising performance in titanium alloy machining, significantly reducing friction and wear, minimizing thermal damage, and enhancing surface quality [91].

### 5.3. Coating Applications

GO-based coatings have emerged as effective tribological solutions for improving surface durability and wear resistance. GO can be incorporated into polymeric, ceramic, and metallic coatings, where it acts as a self-lubricating agent and reduces surface friction [69]. GO coatings exhibit excellent adhesion, corrosion resistance, and mechanical strength, making them suitable for applications in aerospace, automotive, and biomedical industries. Furthermore, the hydrophilic nature of GO allows for the development of water-based coatings, which are environmentally friendly alternatives to conventional oil-based lubricants. The reduction of GO to reduced GO (rGO) within coatings further enhances its tribological performance by increasing conductivity and mechanical stability [92,93]. In addition to lubrication, GO-based coatings serve as anti-corrosion and anti-wear protective layers in harsh environments. The layered structure of GO provides a barrier against moisture and oxidation, preventing substrate degradation [67]. Researchers are exploring hybrid GO coatings combined with nanoparticles or polymer matrices to enhance their durability and adaptability to extreme conditions, paving the way for advanced multifunctional coatings [94]. Table 10 presents various GO-based coating systems and their corresponding improvements in friction and wear, highlighting GO’s role in enhancing surface performance in protective and functional coatings.

### 5.4. Self-Lubricating Properties in Composites

GO-reinforced composites exhibit self-lubricating behavior, making them highly suitable for applications requiring low friction and wear resistance [97]. The incorporation of GO into polymeric, ceramic, and metallic matrices enhances the mechanical and tribological properties of composites. In polymer-based composites, GO improves thermal stability, mechanical strength, and wear resistance while maintaining flexibility. In ceramic and metal matrix composites, GO serves as an effective reinforcement, providing enhanced toughness and self-lubricating effects under varying load conditions. The layered structure of GO ensures continuous lubrication, reducing material degradation and extending the lifespan of composite components [79].

The ability of GO to modify composite surface properties also enhances load distribution and impact resistance. GO-based self-lubricating composites have shown promising results in applications such as bearings, gears, and structural components where conventional lubricants are ineffective. Further developments in GO composite processing techniques aim to optimize dispersion and interfacial bonding, leading to enhanced tribological performance and longevity.

### 5.5. Performance in Extreme Environments

GO demonstrates promising tribological performance in extreme environments, including high-temperature, vacuum, and corrosive conditions. Unlike traditional lubricants that degrade under extreme thermal and oxidative stress, GO maintains its structural integrity and lubricating properties. Studies have reported that GO exhibits excellent thermal stability, withstanding temperatures above 300 °C without significant degradation [98]. In vacuum environments, where conventional lubricants fail due to evaporation, GO provides stable lubrication through its solid-phase interactions. Additionally, GO-based lubricants show superior corrosion resistance, making them ideal for applications in marine, aerospace, and nuclear industries. The ability to functionalize GO further enhances its adaptability to extreme conditions, enabling the development of next-generation tribological materials [99]. Furthermore, GO’s excellent chemical stability allows it to function in aggressive media such as acids, bases, and radiation-exposed environments [100]. Its role as a protective tribological material in such conditions ensures minimal wear and extended service life for critical components. Future research is focused on enhancing GO’s oxidation resistance and improving its compatibility with other extreme-environment materials to develop more resilient lubrication solutions. Table 10 illustrates how GO maintains its tribological effectiveness under extreme conditions such as high temperatures, high loads, and aqueous environments, supporting its use in demanding applications. Table 11 summarizes the performance of GO in extreme environments, highlighting various material systems and their tribological improvements.

Overall, GO’s exceptional tribological properties and versatility make it a key material for advancing lubrication technologies across various industries. Further research into functionalization, dispersion stability, and large-scale synthesis will enhance its potential for commercial applications.

## 6. GO Composites in Tribology

### 6.1. Polymer-Based Composites

Polymer-based composites reinforced with GO have gained considerable attention for tribological applications due to their lightweight nature, corrosion resistance, and enhanced self-lubrication [102]. GO is often incorporated into polymers such as polytetrafluoroethylene (PTFE), epoxy resins, and polyimides to improve wear resistance and friction reduction. The layered structure of GO provides an effective lubricating mechanism by forming a protective film between sliding surfaces, minimizing direct contact and reducing wear [103]. Studies have shown that GO-reinforced polymer composites exhibit a lower coefficient of friction (COF) and higher durability compared to conventional polymer matrices [104]. Additionally, the dispersion of GO within polymer matrices influences the mechanical and thermal stability of the composite. Proper dispersion techniques, such as ultrasonic exfoliation or solution blending, ensure uniform distribution of GO, leading to consistent tribological performance. Moreover, polymer-based GO composites have been applied in aerospace, automotive, and biomedical applications where enhanced durability and reduced friction are critical. The ability of GO to modulate viscoelastic properties also contributes to energy dissipation during dynamic loading, further improving the longevity of polymer-based components [105].

### 6.2. Metal Matrix Composites

Metal matrix composites (MMCs) incorporating GO have demonstrated superior mechanical and tribological properties, making them suitable for high-load applications. GO can be added to metals such as aluminum, copper, and titanium to enhance hardness, thermal stability, and wear resistance [106]. The presence of GO in MMCs contributes to improved load-bearing capabilities by reducing adhesive wear and acting as a solid lubricant at the sliding interface. Additionally, GO’s ability to form a tribofilm during sliding enhances lubrication efficiency, reducing energy losses due to friction [107]. Furthermore, the interfacial bonding between GO and the metal matrix is a key factor influencing the composite’s overall performance. GO’s oxygen-containing functional groups facilitate strong interfacial adhesion, leading to enhanced mechanical integrity. In applications such as gears, bearings, and cutting tools, GO-based MMCs have demonstrated prolonged service life and reduced material degradation under extreme operating conditions. The electrical conductivity of metal-based GO composites also makes them advantageous for use in electronic and thermal management systems where wear resistance and efficient heat dissipation are required [108].

Recent studies have also explored graphene (G) and GO integration with nickel-based superalloys, highlighting substantial improvements in wear resistance, mechanical strength, and overall tribological performance. Nickel–superalloy composites reinforced with G or GO demonstrate enhanced hardness, reduced abrasive wear, and increased load-bearing capabilities due to effective reinforcement and tribofilm formation during tribological contact. Such nickel–G/GO composites are particularly advantageous in high-temperature, high-stress applications, including aerospace engine components, turbine blades, and high-performance bearings, where mechanical integrity and friction control under extreme conditions are critical [109,110].

### 6.3. Ceramic Matrix Composites

Ceramic materials are known for their high hardness and thermal stability, but their brittleness limits their tribological applications. The incorporation of GO into ceramic matrix composites (CMCs) enhances fracture toughness, wear resistance, and self-lubrication. Common ceramic matrices used with GO include alumina (Al_2_O_3_), zirconia (ZrO_2_), and silicon carbide (SiC) [111]. The presence of GO in these composites helps in reducing grain boundary friction and suppressing crack propagation, leading to improved durability in extreme conditions.

Another advantage of GO in CMCs is its role in modifying thermal conductivity and oxidation resistance. The addition of GO enhances the ability of ceramics to withstand high-temperature environments while maintaining structural integrity. This makes GO-reinforced ceramic composites highly suitable for aerospace, biomedical implants, and energy applications. The formation of a lubricating tribo-layer on ceramic surfaces further aids in reducing surface roughness and improving the longevity of components subjected to repeated frictional contact [112].

Various GO-based composites exhibit enhanced tribological properties depending on the matrix material. Table 12 summarizes the composite types and their corresponding improvements in tribological performance.

### 6.4. Hybrid Composites

Hybrid composites integrate GO with multiple reinforcing agents, such as carbon nanotubes (CNTs), boron nitride (BN), or other nanomaterials, to achieve synergistic improvements in tribological performance. These composites exhibit enhanced wear resistance, reduced friction, and improved thermal stability due to the combined effects of multiple reinforcing phases [117]. Hybrid composites are particularly effective in demanding applications where conventional materials fail to provide adequate lubrication and durability. Table 13 gives a few examples of hybrid composites and their key benefits.

The design of hybrid composites requires careful selection of secondary reinforcement materials to maximize their synergistic effects. CNTs and BN, for example, complement GO’s lubricating properties while reinforcing mechanical strength. These multi-phase composites are extensively studied for applications in extreme environments, such as space exploration and high-performance machinery. By leveraging the combined advantages of GO and other nanomaterials, hybrid composites provide an optimized balance of wear resistance, friction control, and load-bearing capacity.

## 7. Mechanisms of Tribological Performance Improvement

### 7.1. Mechanisms of Wear Reduction

**Figure 4:**
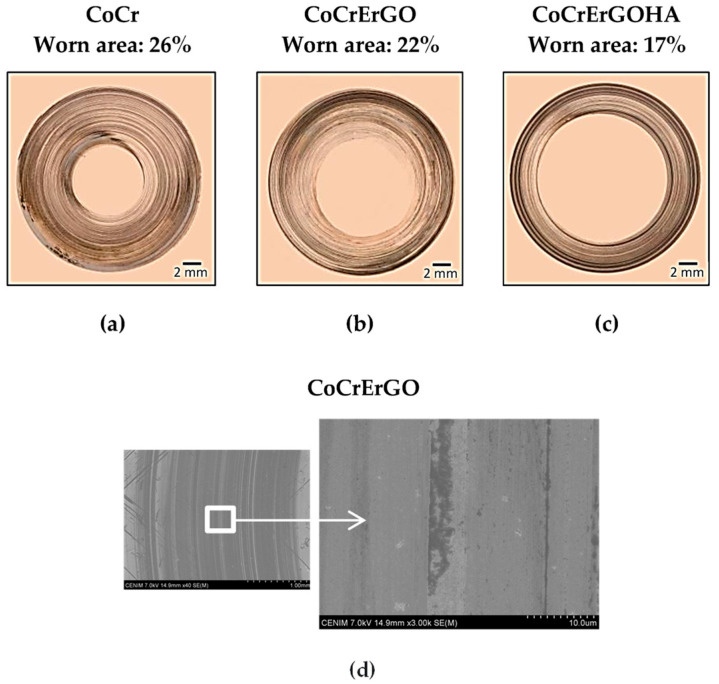
Optical microscopy images of the worn tracks after tribo-corrosion tests in CoCr (**a**), CoCrErGO (**b**), and CoCrErGOHA (**c**) after 30,000 m of sliding distance. Scanning electron microscopy image of the worn track after tribo-corrosion testing in CoCrErGO (**d**) after 30,000 m of sliding distance [126].

Wear reduction in GO-based composites is primarily achieved through multiple mechanisms, including (a) Formation of a protective film: GO forms a thin lubricating layer on the contact surfaces, reducing direct asperity contact and minimizing abrasive wear [122]; (b) Load bearing and stress distribution: The high mechanical strength of GO allows for effective load distribution, reducing localized stress concentrations that lead to wear [123]; (c) Enhanced hardness and toughness: GO improves the hardness and toughness of composite materials, making them more resistant to material loss due to sliding or impact [124]; (d) Self-Healing properties: In some cases, GO’s chemical functionality enables self-repair of minor surface damage, further enhancing wear resistance [125]. The “self-healing” behavior observed with GO arises from its ability to form dynamic, adaptive tribofilms under contact stress. During sliding, GO nanosheets can migrate, reorient, or even fragment to fill micro-cracks and grooves, effectively restoring a lubricating barrier that prevents direct surface contact. Localized frictional heat also helps align or compact GO layers, enhancing this self-replenishing effect. Such mechanisms maintain low friction and protect against progressive wear, even after partial tribofilm disruption [75]. Figure 4 shows the effect of GO composite film on surfaces and its tribological study results. Additionally, GO interacts with the matrix material to modify wear behavior based on the surrounding environment. Under dry conditions, GO’s ability to absorb and retain ambient moisture helps in maintaining lubricity. In lubricated systems, GO disperses effectively within liquid lubricants, providing additional anti-wear protection. These adaptive characteristics make GO a versatile reinforcement for various tribological applications.

### 7.2. Role in Friction Control

GO plays a crucial role in friction control through several key mechanisms, as follows: (a) Layered structure and interlayer sliding: The layered structure of GO allows easy interlayer sliding, acting as a solid lubricant that reduces friction at the interface [127]; (b) Surface functionalization: The functional groups on GO influence adhesion and interaction with mating surfaces, thereby enhancing frictional performance [128]; (c) Thermal stability and heat dissipation: GO helps in dissipating heat generated during frictional contact, preventing thermal degradation of composite materials [129]; (d) Adaptive lubrication: In dynamic conditions, GO exhibits adaptive lubrication behavior, where it responds to varying loads and sliding conditions by adjusting its lubricating efficiency [130].

**Figure 5:**
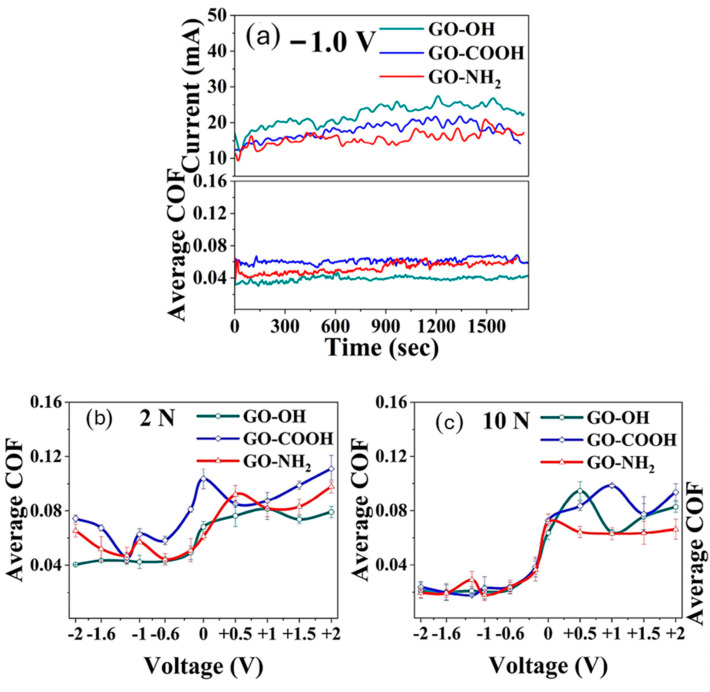
(**a**) Current and COF changes in the composite solutions under −1.0 V; (**b**,**c**) friction test results of functionalized GO additives with different voltage stimulation at (**b**) 2 N and (**c**) 10 N [131]. Reproduced under the terms of the Creative Commons Attribution License (CC BY).

Electric vehicles have recently become more widely available in the market. Therefore, the study of friction behavior by considering the effect of electrification is crucial for deriving the effect on tribological properties. Figure 5 illustrates the tribological behavior of functionalized GO with OH, COOH, and NH_2_ groups under varying electrochemical potentials and mechanical loads. At a constant voltage of –1.0 V (Figure 5a), GO–NH_2_ exhibits the lowest coefficient of friction (COF), followed by GO–OH and GO–COOH, indicating better lubricity and electrochemical stability. Under a 2 N load (Figure 5b), all GO variants maintain low COF at negative voltages, but COF increases at positive voltages, with GO–NH_2_ showing the most stable performance. This trend continues under a 10 N load (Figure 5c), where GO–NH_2_ consistently demonstrates superior tribological behavior across voltages. Overall, the results suggest that the type of functional group significantly influences the frictional properties of GO, with GO–NH_2_ offering the most effective lubrication, particularly under electrochemical control and higher mechanical loads. Furthermore, the friction control properties of GO are influenced by its concentration, dispersion, and compatibility with the base material. When properly integrated, GO not only reduces static and kinetic friction but also minimizes stick-slip behavior, enhancing overall operational efficiency.

### 7.3. Nanoscale Mechanisms of Friction and Wear Reduction

The tribological performance of GO at the nanoscale is governed by a combination of mechanical, physical, and chemical mechanisms that operate synergistically during sliding contact. These mechanisms reduce both friction and wear in materials subjected to mechanical loading.

One of the primary nanoscale mechanisms is interlayer sliding. GO consists of two-dimensional sheets with a layered structure that enables facile shear between adjacent layers under applied load [21,22]. This layered architecture acts as an atomically smooth interface, reducing shear resistance, which translates to a lower coefficient of friction. Similar to graphite and MoS_2_, the weak van der Waals forces between layers in GO allow slippage, thereby minimizing energy dissipation during sliding motion [24,25,73].

Another critical mechanism is tribofilm formation. During sliding contact, GO nanosheets can orient along the direction of motion and form a compact, adherent layer at the interface [75]. This layer acts as a protective barrier between mating surfaces, preventing direct metal-to-metal contact and reducing adhesive and abrasive wear. Tribofilms formed from GO are known to be resilient and can adapt dynamically to changing loading conditions, thereby prolonging component life [21,24].

Tribochemical transformation is also significant at the nanoscale. Under tribological stress and localized heat, the oxygen-containing functional groups in GO (such as carboxyl, hydroxyl, and epoxy) can undergo reactions that result in the partial reduction of GO into rGO or carbon-rich films [39,60]. These tribo-induced chemical reactions modify surface energy and contribute to the formation of lubricious carbonaceous layers that reduce friction and surface degradation [73].

Self-healing behavior is another observed nanoscale phenomenon with GO-based systems. As GO sheets experience delamination or wear during operation, newly exposed GO fragments can migrate into the contact zone and replenish worn-out regions [75]. This mechanism maintains a continuous lubricating interface and stabilizes friction performance over long durations. The flexibility and structural adaptability of GO nanosheets support this regeneration process [21,75].

Additionally, GO’s hydrophilic functional groups can interact with ambient humidity, forming a thin boundary lubrication film due to absorbed water molecules [28]. This effect is particularly pronounced in humid environments, where moisture-mediated lubrication contributes to reduced interfacial friction. The presence of oxygen groups also enhances adhesion to metallic or ceramic substrates, improving tribofilm stability [16,24,28].

Finally, the presence of sp^2^-hybridized carbon domains in GO contributes to electron cloud repulsion, which reduces adhesive interactions at the interface and minimizes energy losses during sliding. This effect becomes critical in dry or vacuum environments, where traditional liquid lubrication is ineffective [21,76].

Together, these nanoscale mechanisms, including interlayer shear, tribofilm formation, tribochemical transformation, self-healing, and surface charge interactions, contribute to GO’s superior performance as a friction-reducing and anti-wear additive in solid and liquid lubrication systems.

## 8. Synthesis and Functionalization Techniques

### 8.1. Chemical Methods

GO is typically synthesized through chemical oxidation of graphite using methods such as the Hummers [132], modified Hummers [133], or Tour method [134]. These methods involve the use of strong oxidants such as potassium permanganate (KMnO_4_) and sulfuric acid (H_2_SO_4_), which introduce oxygen-containing functional groups (hydroxyl, carboxyl, and epoxy) onto the graphene sheets. These groups are responsible for GO’s hydrophilicity and reactivity, making it highly dispersible in aqueous solutions and enabling further functionalization. Post-synthesis chemical reduction of GO to reduced GO (rGO) using agents such as hydrazine hydrate or ascorbic acid is also widely practiced to tune the electrical and mechanical properties for specific applications [135,136].

Functionalization through chemical grafting with polymers, surfactants, or organic molecules is another widely adopted method to improve dispersion and compatibility with various matrices [137,138]. This surface modification enhances GO’s interaction with polymeric, ceramic, or metallic systems, leading to improved load transfer and tribological behavior in composite materials. While reduction and functionalization are essential for tailoring GO’s compatibility and properties, they also introduce notable trade-offs. Excessive chemical reduction, for instance, can restore electrical conductivity and strengthen interlayer adhesion but often compromises dispersion stability, leading to agglomeration. Similarly, covalent functionalization improves interfacial bonding in composites but can disrupt the sp^2^ carbon network, reducing mechanical strength and altering intrinsic lubricity. Many functionalization routes also involve multi-step chemistries that may be costly or difficult to scale for industrial applications. Balancing these effects is critical for achieving optimized performance in tribological systems [21,24,26].

### 8.2. Physical Methods

Physical synthesis techniques for GO and its functionalized forms include methods such as mechanical exfoliation [139], ultrasonication [140], and ball milling [141]. These approaches are often used in combination with chemical methods to achieve a high surface area and few-layer GO sheets. For instance, ultrasonication helps break down graphite oxide into smaller flakes and improves the homogeneity of GO dispersions.

Thermal treatment is another important physical technique used to reduce GO and tailor its structural and tribological properties. Techniques such as thermal annealing in an inert atmosphere can selectively remove oxygen functional groups and enhance the crystallinity and conductivity of GO [142]. Plasma treatment and irradiation techniques have also been explored to introduce specific functional groups or to improve GO’s compatibility with various substrates [143,144].

### 8.3. Challenges in Large-Scale Production

Scaling up the synthesis and functionalization of nanomaterials from laboratory to industrial levels presents a series of complex technical and logistical challenges. Many of the most effective lab-scale techniques, such as hydrothermal synthesis, sol–gel processing, chemical vapor deposition, and freeze-drying, are batch-based and difficult to adapt for continuous, high-throughput production. This limitation in scalability becomes more evident when attempting to manufacture uniform materials at larger volumes, as these processes often require precise control over temperature, pressure, pH, and precursor concentrations. Furthermore, cost and resource intensity are major barriers, with many synthesis methods depending on expensive reagents, high-purity solvents, specialized equipment, and energy-intensive conditions. Environmental concerns also arise due to the frequent use of hazardous chemicals and the generation of toxic waste, posing additional hurdles in meeting sustainability goals and industrial regulations [145].

**Figure 6:**
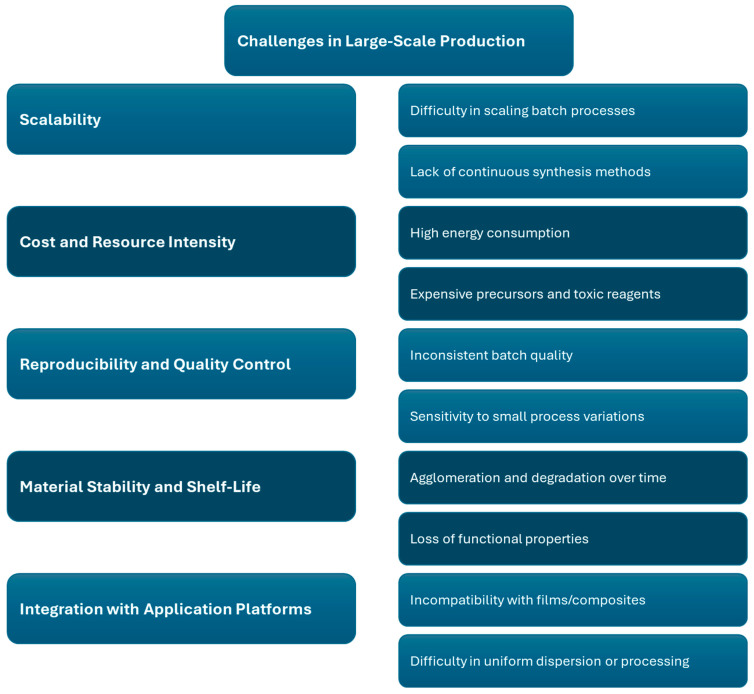
Challenges of the large-scale production of GO.

Beyond production logistics, several material-specific challenges must be addressed to ensure successful commercialization. One major issue is reproducibility and quality control [146]. Nanomaterials are highly sensitive to minor fluctuations in processing conditions, which can lead to inconsistent particle size, surface functionalization, porosity, and morphology—factors that critically affect their end-use performance. Additionally, maintaining material stability and shelf-life during storage and transport is crucial, as some nanostructures tend to agglomerate, oxidize, or degrade over time, especially in ambient conditions [147]. A further challenge lies in integration with application platforms. Whether used in composites, coatings, biomedical scaffolds, or electronic devices, nanomaterials must often be transformed into specific formats without compromising their properties. This demands compatibility with secondary materials, robust processing techniques, and often post-treatment steps such as curing or crosslinking. Coupled with regulatory constraints, economic viability, and the lack of standardization, these factors collectively hinder the widespread adoption of nanomaterials despite their tremendous potential [148,149]. Overcoming these obstacles requires interdisciplinary collaboration to develop greener, scalable, and application-oriented synthesis strategies. Figure 6 provides an overview of the challenges of large-scale production.

## 9. Challenges and Limitations in Graphene Oxide Applications

### 9.1. Stability Under Operational Conditions

GO’s performance is highly influenced by its stability in changing environmental and operational conditions. GO is thermally unstable at high temperatures; decomposition and loss of oxygen functional groups usually occur around 150–200 °C, which limits its use in elevated temperature applications [150]. The functional groups that make GO versatile make it chemically reactive in low- or high-pH environments. GO can undergo unwanted transformations that affect its consistency and function. Another concern is photodegradation, as exposure to UV or visible light can cause GO to partially reduce, leading to color changes, loss of hydrophilicity, and altered conductivity [151]. In aqueous dispersions, GO is initially stable, but it may become aggregated or sedimented over time, so its shelf-life and processability will be compromised. In biomedical environments, variations in ionic strength, pH, or enzymatic activity can lead to unpredictable degradation or interaction with biomolecules, raising concerns about their long-term biocompatibility and safety. Table 14 gives a brief idea about GO’s stability under various operational conditions.

### 9.2. Cost and Scalability

Even though GO is easier to produce than pristine graphene, its mass production is limited by several factors. The most common synthesis route, the modified Hummers method, relies on concentrated acids and strong oxidizers, which are hazardous, corrosive, and environmentally taxing. This method will need careful waste management, as it limits scalability with safety concerns [157]. Moreover, batch-to-batch variability is still an issue, as oxidation level, flake size, and purity differences can affect downstream usage. Post-synthesis steps such as washing, filtration, dialysis, or centrifugation are resource-intensive and time-consuming, reducing the efficiency of large-scale production. Also, the lack of industrial infrastructure for standard and high-throughput GO synthesis, along with surging environmental regulations, adds to the cost. While green and continuous-flow synthesis methods are being explored [158], they are not yet industrially mature, which keeps GO production expensive and less viable for cost-sensitive industries.

In addition to synthesis-related challenges, the functionalization of GO at an industrial scale further complicates cost and reproducibility. Covalent and non-covalent modification techniques—essential for enhancing GO’s dispersibility and tribological compatibility—require tightly controlled reaction environments. However, batch-to-batch variations in GO flake size, defect density, and oxygen group distribution make it difficult to achieve uniform functionalization across large volumes [70]. Such inconsistencies affect the downstream performance of GO in tribological systems, often requiring extensive post-functionalization purification and characterization, which adds to the processing time and cost [159]. Moreover, many functionalization reagents, such as silanes or organic amines, are moisture-sensitive and degrade in storage, reducing scalability. Despite research into greener or in situ modification approaches [160], commercially viable, high-throughput functionalization methods with consistent output remain underdeveloped, making GO-based lubricant additives expensive and difficult to standardize for industrial use.

Despite promising lab-scale results, the lack of standardized tribological testing protocols for GO-based additives remains a major bottleneck for industrial adoption. Reported friction and wear performance vary widely due to differences in test loads, contact configurations, surface materials, lubrication conditions, and environmental factors (e.g., humidity, temperature) [56,113]. This variability complicates meaningful comparison across studies and hinders reproducibility. Standardized test methodologies, such as specifying test geometry (ball-on-disk, pin-on-disk), normal load, sliding speed, and temperature ranges, are essential for benchmarking GO additives under application-relevant conditions [159]. Furthermore, long-duration testing and accelerated wear simulation protocols are needed to better evaluate GO’s durability, stability, and performance degradation over time. Establishing such standardized frameworks would not only improve reproducibility but also facilitate regulatory approval and industrial integration of GO-based tribological materials.

### 9.3. Compatibility with Base Materials

A major limitation in the practical application of GO is its compatibility with various base materials used in composites, coatings, and functional systems. GO has a strong tendency to agglomerate or restack due to interlayer van der Waals forces, specifically during solvent evaporation or thermal processing [159]. This can lead to low dispersion within matrices such as polymers, which will result in localized defects or inconsistent mechanical properties. GO’s high surface energy and polar functional groups frequently lead to weak interfacial bonding with hydrophobic substrates, such as polypropylene or epoxy, unless surface modification is done. In a few cases, reactive byproducts from GO synthesis can corrode metal substrates or interfere with the curing process of polymers [25]. Moreover, introducing GO into a matrix can change the system’s mechanical or electrical properties; excessive loading can increase stiffness but reduce toughness or flexibility. Thus, accomplishing chemical and mechanical compatibility without compromising the host material’s integrity remains a major challenge in advancing GO-based technologies.

## 10. Future Directions and Emerging Trends

When GO is introduced with base materials such as composites, coatings, and functional systems, there will be compatibility issues, resulting in limitations in practical usage. GO has an inherent tendency to agglomerate, which results in poor dispersion within matrices such as polymers, resins, or solvents. This can lead to localized structural defects, inconsistent mechanical performance, and compromised reliability of the final material. Proper dispersion is crucial as it influences properties like tensile strength, elasticity, electrical conductivity, and thermal stability.

This agglomeration can result in poor dispersion within matrices such as polymers, resins, or solvents, which can lead to structural defects, affect mechanical performance, and reliability in the final composite material [160]. One of the major hurdles in the long-term implementation of GO-based lubricants is agglomeration caused by van der Waals attractions and π–π stacking between nanosheets. These interactions increase over time, especially in static or low-flow conditions, leading to sedimentation or clogging in industrial systems. This risk becomes more pronounced under high-temperature or cyclic thermal environments, where changes in fluid viscosity and oxidation state can further destabilize GO dispersions. To counteract this, various functionalization techniques, such as grafting hydrophilic or hydrophobic groups, have been employed to enhance colloidal stability and interfacial compatibility with base oils [70]. However, these strategies are often sensitive to pH, temperature, and shear rate, limiting their robustness in field-scale applications. Achieving long-term dispersion without compromising tribological properties remains an open challenge that must be addressed through the development of multifunctional surfactants or core–shell structured additives that can dynamically stabilize GO nanosheets during operation.

The compatibility between GO and multiple base materials is determined by surface chemistry. Since GO has high surface energy and consists of polar oxygen-containing functional groups (e.g., hydroxyl, epoxy, and carboxyl), robust interfacial adhesion with hydrophobic substrates such as polypropylene, polyethylene, or epoxy resin can be challenging. This conflict in surface energies can lead to weak interfacial bonding, which can reduce load transfer efficiency in structural composites and weaken durability under mechanical stress [161]. To eradicate these issues, large surface modifications such as salinization, polymer grafting, or chemical reduction are mostly required to ensure the required wettability and compatibility, which increases production complexity and cost.

The GO synthesis process poses complications such as interfacial challenges, residual acidity, and reactive chemical byproducts. These residuals can cause corrosion or degradation when GO is combined with metallic substrates, therefore compromising structural integrity and long-term stability of metal–GO interfaces [162]. Acidic residues can adversely affect polymer curing processes by interfering with crosslinking mechanisms, reducing the mechanical strength, durability, and thermal stability of polymer GO composites. Also, if we incorporate GO into composite systems, it will alter mechanical or electrical behaviors.Excessive GO loading can inadvertently increase material stiffness, but at the expense of reducing toughness, ductility, and flexibility—critical attributes in many practical engineering applications. In electronic composites, improper dispersion or integration of GO sheets can lead to hindered electron transfer pathways, severely diminishing electrical conductivity and overall device efficiency [163]. Thus, striking an optimal balance between loading, dispersion, and functionality without adversely impacting the host material’s fundamental properties remains an ongoing challenge.

Overall, to overcome these compatibility limitations, it is necessary to use strategic material engineering and innovative approaches, as well as optimizing processing techniques and following strict quality control methods. Addressing these challenges is crucial to realizing the commercial and industrial potential use for GO-based technologies.

## 11. Conclusions

GO has emerged as a promising material for enhancing tribological performance across various applications, including lubricants, coatings, and composites. Studies discussed in this manuscript have demonstrated that adding GO in small concentrations (typically 0.05 to 2 wt%) can lead to significant improvements. In lubricants, GO added at 0.1–0.5 wt% effectively forms a protective tribofilm, resulting in a friction reduction up to 50% and a wear rate decrease by 40–70%, depending on the load and operating environment. In coating applications, GO incorporation improves adhesion, corrosion resistance, and self-lubrication; coatings with 1–2 wt% GO have shown up to 60% improvement in wear resistance in harsh conditions. Similarly, in GO-reinforced composites, whether polymeric, ceramic, or metallic, GO concentrations ranging from 0.5 to 1.5 wt% enhance load-bearing capacity and reduce direct asperity contact, contributing to 30–60% reductions in friction and wear. The layered structure, surface functionality, and high mechanical strength of GO are central to these enhancements, allowing GO to serve as a solid lubricant, stress distributor, and tribofilm former. However, challenges such as agglomeration, compatibility with base matrices, and scalability of production remain critical barriers. Addressing these through improved functionalization and dispersion strategies will be essential for translating GO’s laboratory success into industrial applications.

## Figures and Tables

**Table 1:** Physical properties of GO and its values.

Property	Approximate Value	Reference
Surface Area	~2630 m^2^/g (specific surface area)	[36]
Functional Groups	Epoxides, hydroxyls, carboxyls (varies based on GO preparation)	[37]
Mechanical Strength	Tensile strength up to ~130 GPa (similar to graphene)	[38]
Self-lubricating Properties	Effective in reducing friction and wear	[35]
Wear Resistance	Improved, depending on application and GO concentration	[24]
Thermal Stability	Stable up to ~200 °C to ~300 °C	[39]
Electrical Conductivity	Low conductivity (but tunable by reduction processes)	[40]
Hydrophilicity	High (due to oxygenated functional groups)	[41]

**Table 2:** Structural properties of GO and its values.

Structural Property	Approximate Value	Reference
Layer Structure	Single-layer (monolayer) or few layers	[43]
Lattice Arrangement	Hexagonal (similar to graphene)	[44]
Thickness	~1 nm (monolayer)	[43]
Oxygen Content	20–50% by weight (depends on degree of oxidation)	[45]
Defect Density	High (due to oxygen functional groups and oxidation)	[46]
Interlayer Spacing	~0.8–1.0 nm (varies with oxidation level)	[47]

**Table 3:** Chemical properties of GO and its values.

Chemical Property	Approximate Value	Reference
Chemical Reactivity	High (due to the presence of oxygenated groups)	[48]
pH (in aqueous dispersion)	~3–6 (varies based on the degree of oxidation)	[49]
Surface Charge	Negative (due to carboxyl groups and hydroxyls)	[50]
Degree of Oxidation	20–50% (tunable depending on the preparation method)	[51]
Reduction Potential	Can be reduced to improve conductivity (e.g., reduced GO has better electrical properties)	[44]
Solubility	Soluble in water and polar solvents	[52]

**Table 4:** Mechanical properties of GO and its values.

Mechanical Property	Approximate Value	Reference
Tensile Strength	100–200 MPa (GO films)	[54]
Elastic Modulus	5–40 GPa (varies with oxidation)	[55]
Fracture Toughness	Lower than graphene, brittle nature	[56]
Interlayer Adhesion	High (due to hydrogen bonding) compared to graphene	[57]

**Table 5:** Thermal properties of GO and its values.

Property	Approximate Value	Reference
Thermal Stability	Stable up to ~200–300 °C	[39]
Decomposition Temperature	~200–300 °C (due to oxygen group removal)	[62]
Thermal Conductivity	~0.14–2.87 W/m·K (varies with oxidation)	[63]
Electrical Conductivity	Insulating (can be restored by reduction)	[60]

**Table 6:** Tribological application area and its benefits and applications.

Application Area	Key Benefits	Example Applications	Reference
Solid Lubricant Properties	Low shear strength, protective film formation, self-healing behavior	High-precision mechanical systems, aerospace components	[21]
Additives in Lubricants	Forms stable tribofilms, reduces wear and friction, enhances thermal stability	Automotive lubricants, industrial machinery oils	[68]
Coating Applications	Enhances wear resistance, corrosion protection, and adhesion	Aerospace coatings, biomedical implants, anti-corrosion coatings	[69]
Self-Lubricating Properties in Composites	Improves mechanical properties, reduces material degradation, enhances load distribution.	Bearings, gears, structural components	[70]
Performance in Extreme Environments	Stable at high temperatures, vacuum lubrication, corrosion resistance	Marine environments, space applications, nuclear reactors	[71]

**Table 7:** Graphene oxide as a solid lubricant—material pairs and tribological performance.

Material Pair	Friction Reduction	Wear Reduction	Test Conditions	Reference
Stainless Steel–Stainless Steel (with Graphene-ZnO-PVDF composite)	Up to 90%	Up to 90%	Load: 15 N normal load. Distance: 450 m sliding. Environment: Room temperature.	[77]
Stainless Steel–Stainless Steel (with MoS_2_–GO hybrid coating)	Excellent performance in various environments	Survived 44 km of sliding	Load: 1, 3, 5, 7, 9 N. Environment: ambient atmospheric conditions, dry nitrogen, and in a high vacuum.	[75]
Silicon–Silicon (with GO/PDDA multilayer films)	Lower friction coefficients with increased layers	Enhanced wear resistance with increased layers	Distance: Reciprocating at a distance of 5 mm. Load: of 0.1, 0.2, 0.3, and 0.4 N. Environment: Ambient conditions of 20 °C and 40–50% relative humidity.	[78]

**Table 8:** Comparison of graphene oxide (GO) with common solid lubricants.

Property/Feature	Graphene Oxide (GO)	Molybdenum Disulfide (MoS_2_)	Graphite	Hexagonal Boron Nitride (h-BN)	PTFE
Structure	2D oxidized graphene sheets [21,22]	Layered crystalline (Mo–S–Mo) [76]	Layered carbon sheets [24]	Layered hexagonal structure [25]	Linear fluoropolymer [21]
Wear Resistance	High (forms tribofilm) [21,22,75]	High but degrades in air [74]	Moderate [24,76]	High [25]	Moderate-to-high [21,23]
Thermal Stability (°C)	200–300 °C (starts decomposing) [39,60]	Stable up to 400 °C in inert, <300 °C air [74]	~500 °C inert, ~300 °C air [24]	Up to 900 °C inert [25]	Up to 260 °C [21]
Oxidation Resistance	Moderate-to-good [21,24]	Poor above 250 °C in air [74]	Moderate [24]	Excellent [25]	Good [21]
Chemical Reactivity	High, easily functionalized [16,21]	Inert, but oxidizes in moist air [74]	Inert [24]	Stable [25]	Inert [21]
Lubrication in Humid Air	Excellent (hydrophilic) [24,28]	Poor (sensitive to moisture) [74]	Moderate [24]	Stable [25]	Stable [21]

**Table 9:** Graphene oxide-based liquid lubricant: material pairs and tribological performance.

Material Pair	GO Concentration	Friction Reduction	Wear Reduction	Test Conditions	Reference
Steel–Steel (PAO 6 base oil)	0.5 wt%	Up to 30%	Not specified	Load of 2 N Distance: sliding stroke are 0.4 Hz and 3 mm Environment: Not specified	[84]
Magnesium Alloy–Steel (Water-based nanofluid)	0.5 wt%	77.50%	90%	Load: 1, 3, 5, 8 N Distance: 0.08 m/s for 0.5 h Environment: Room temperature	[85]
Steel–Steel (Engine oil)	0.02 wt%	5%	3%	Load: 60.5 N Distance: Sliding speed and stroke 0.055 m/s and 8 mm Environment: Elevated temperature, 100 deg	[86]

**Table 10:** Graphene oxide-based coatings: types and tribological performance.

Coating Type	GO Concentration	Friction Reduction	Wear Reduction	Test Conditions	Reference
Micro-Arc Oxidation (MAO) Coating on Ti-6Al-4V Alloy	5 g/L	Reduced from 0.47 to 0.35 (~25%)	Not specified	Load: 3 N Distance: 100 rpm, with a diameter of 6 mm Environment: 20 ± 1 °C.	[95]
Polyurethane (PU) Composite Coating Reinforced with Functionalized GO	0.25–0.5 wt%	Not specified	Improved wear resistance	Load of 3 N, Distance: 20 min and wear track length of 5 mm. Environment: Ambient	[96]
Layer-by-Layer Assembled GO/PDDA Multilayer Films on Silicon Substrate	Not specified	Decreased with more layers	Increased with more layers	Loads: 0.1, 0.2, 0.3, and 0.4 N Distance: Reciprocating 5 mm Environment: 20 °C and 40–50% relative	[78]

**Table 11:** Graphene oxide performance in extreme environments: materials and tribological performance.

Environment	Materials Used	GO Concentration	Friction Reduction	Wear Reduction	Test Conditions	Reference
High Temperature (up to 400 °C)	PTFE/GO Composite Coating	15 vol%	Reduced to 0.1	0.65 × 10^−9^ mm^3^/N·m	Load: 5 N, a Distance: Sliding speed of 4 mm s−1 and a stroke of 4 mm	[101]
High-Load Conditions	Graphene-ZnO Composite Film	Not specified	Up to 90%	Up to 90%	Load: 15 N normal load Distance: 450 m sliding	[77]
Water-Based Nanofluid Environment	0.5 wt% GO in Water (Mg Alloy–Steel Pair)	0.5 wt%	77.50%	90%	Load: 1, 3, 5, 8 N Distance: 0.08 m/s for 0.5 h Environment: Room temperature	[85]

**Table 12:** Summary of GO-based composites and their tribological applications.

GO Composite Type	Matrix Material	Tribological Improvements	Mechanism	References
GO–Polymer Composites	Epoxy, Nylon, PTFE, etc.	Reduced friction and wear rate	Uniform dispersion and strong interfacial bonding due to GO’s functional groups	[113]
GO–Metal Matrix Composites (MMCs)	Aluminum, Copper, etc.	Enhanced load-carrying capacity and wear resistance	GO acts as a barrier and provides solid lubrication under sliding	[114]
GO–Ceramic Composites	Al_2_O_3_, Si_3_N_4_, TiO_2_	Improved hardness, wear resistance, and reduced crack propagation	GO bridges microcracks and enhances grain boundary strength	[115]
GO–Ceramic	SiC (3D GO–CNT hybrid)	Improved hardness, wear resistance, reduced friction and crack propagation under dry sliding	Hybrid GO–CNT network tribofilm; crack bridging and shear dissipation	[116]

**Table 13:** Hybrid composites and their benefits.

Hybrid Composite Type	Secondary Reinforcement	Key Benefits	Reference
GO-CNT Composites	Carbon Nanotubes (CNTs)	Enhanced mechanical strength, improved thermal conductivity, and reduced friction	[118]
GO-BN Composites	Boron Nitride (BN)	Increased lubrication efficiency, superior high-temperature stability	[119]
GO-MoS_2_ Composites	Molybdenum Disulfide (MoS_2_)	Excellent solid lubrication, enhanced anti-wear properties	[120]
GO-SiC Composites	Silicon Carbide (SiC)	Superior hardness, improved oxidation resistance in high-temperature applications	[121]

**Table 14:** GO stability under different operating conditions.

Stability Factor	Challenges/Limitations	Potential Impacts	Reference
Thermal Stability	Decomposition and loss of functional groups at elevated temperatures (~150–200 °C).	Limits usage in high-temperature applications, structural integrity loss, inconsistent performance.	[152]
Chemical Reactivity	Highly reactive oxygen functional groups are prone to reduction or reactions in varying chemical environments.	Unpredictable chemical behavior, impaired consistency, potential toxicity in biomedical uses.	[153]
Photodegradation	Partial reduction and structural damage upon prolonged UV or visible light exposure.	Altered optical and electronic properties, decreased reliability in solar and optical applications.	[154]
Aqueous Dispersion Stability	Tendency to aggregate, sediment, or spontaneously reduce over time in aqueous dispersions.	Shortened shelf-life, inconsistent performance in formulations, difficulties in handling.	[155]
Biological Stability	Degradation or unwanted reactions in biological media (pH shifts, enzymatic activity, ionic strength).	Unpredictable biocompatibility and cytotoxicity, compromised efficacy in biomedical applications.	[156]

## Data Availability

No new data were created or analyzed in this study. Data sharing is not applicable to this article.

## References

[1] Menezes P.L., Nosonovsky M., Ingole S.P., Kailas S.V., Lovell M.R. (2013). Tribology for Scientists and Engineers: From Basics to Advanced Concepts.

[2] Tung S.C., McMillan M.L. (2004). Automotive Tribology Overview of Current Advances and Challenges for the Future. Tribol. Int..

[3] Miyoshi K. (1999). Aerospace Mechanisms and Tribology Technology: Case Study. Tribol. Int..

[4] Mathew M.T., Srinivasa Pai P., Pourzal R., Fischer A., Wimmer M.A. (2009). Significance of Tribocorrosion in Biomedical Applications: Overview and Current Status. Adv. Tribol..

[5] Komanduri R., Hou Z.B. (2001). A Review of the Experimental Techniques for the Measurement of Heat and Temperatures Generated in Some Manufacturing Processes and Tribology. Tribol. Int..

[6] Ralls A.M., Kumar P., Menezes P.L. (2021). Tribological Properties of Additive Manufactured Materials for Energy Applications: A Review. Processes.

[7] Reeves C.J., Menezes P.L., Lovell M.R., Jen T.-C. (2013). The Size Effect of Boron Nitride Particles on the Tribological Performance of Biolubricants for Energy Conservation and Sustainability. Tribol. Lett..

[8] Omrani E., Moghadam A.D., Menezes P.L., Rohatgi P.K. (2016). Influences of Graphite Reinforcement on the Tribological Properties of Self-Lubricating Aluminum Matrix Composites for Green Tribology, Sustainability, and Energy Efficiency—A Review. Int. J. Adv. Manuf. Technol..

[9] Menezes P.L. (2016). Surface Texturing to Control Friction and Wear for Energy Efficiency and Sustainability. Int. J. Adv. Manuf. Technol..

[10] Reeves C.J., Menezes P.L. (2017). Evaluation of Boron Nitride Particles on the Tribological Performance of Avocado and Canola Oil for Energy Conservation and Sustainability. Int. J. Adv. Manuf. Technol..

[11] Sikdar S., Rahman M.H., Menezes P.L. (2022). Synergistic Study of Solid Lubricant Nano-Additives Incorporated in Canola Oil for Enhancing Energy Efficiency and Sustainability. Sustainability.

[12] Kasar A.K., Menezes P.L. (2018). Synthesis and Recent Advances in Tribological Applications of Graphene. Int. J. Adv. Manuf. Technol..

[13] Dorri Moghadam A., Omrani E., Menezes P.L., Rohatgi P.K. (2015). Mechanical and Tribological Properties of Self-Lubricating Metal Matrix Nanocomposites Reinforced by Carbon Nanotubes (CNTs) and Graphene—A Review. Compos. Part B Eng..

[14] Xu M., Liang T., Shi M., Chen H. (2013). Graphene-Like Two-Dimensional Materials. Chem. Rev..

[15] Some S., Xu Y., Kim Y., Yoon Y., Qin H., Kulkarni A., Kim T., Lee H. (2013). Highly Sensitive and Selective Gas Sensor Using Hydrophilic and Hydrophobic Graphenes. Sci. Rep..

[16] Seredych M., Tamashausky A.V., Bandosz T.J. (2010). Graphite Oxides Obtained from Porous Graphite: The Role of Surface Chemistry and Texture in Ammonia Retention at Ambient Conditions. Adv. Funct. Mater..

[17] Jin B., Zhao J., He Y., Chen G., Li Y., Zhang C., Luo J. (2022). High-Quality Ultra-Flat Reduced Graphene Oxide Nanosheets with Super-Robust Lubrication Performances. Chem. Eng. J..

[18] Suo Y., Guo R., Xia H., Yang Y., Zhou B., Zhao Z. (2022). A Review of Graphene Oxide/Cement Composites: Performance, Functionality, Mechanisms, and Prospects. J. Build. Eng..

[19] Liu Y., Ge X., Li J. (2020). Graphene Lubrication. Appl. Mater. Today.

[20] Su Y., Kravets V.G., Wong S.L., Waters J., Geim A.K., Nair R.R. (2014). Impermeable Barrier Films and Protective Coatings Based on Reduced Graphene Oxide. Nat. Commun..

[21] Liang H., Bu Y., Zhang J. (2013). Graphene Oxide Film as Solid Lubricant. ACS Appl. Mater. Interfaces.

[22] Kasar A.K., Xiong G., Menezes P.L. (2018). Graphene-Reinforced Metal and Polymer Matrix Composites. JOM.

[23] Tao F., Salmeron M. (2011). In Situ Studies of Chemistry and Structure of Materials in Reactive Environments. Science.

[24] Sandoz-Rosado E.J., Tertuliano O.A., Terrell E.J. (2012). An Atomistic Study of the Abrasive Wear and Failure of Graphene Sheets When Used as a Solid Lubricant and a Comparison to Diamond-like-Carbon Coatings. Carbon.

[25] Chauhan D.S., Quraishi M.A., Ansari K.R., Saleh T.A. (2020). Graphene and Graphene Oxide as New Class of Materials for Corrosion Control and Protection: Present Status and Future Scenario. Prog. Org. Coat..

[26] Li H., Chen S., Li Z., Feng Y., Zhang M. (2020). Preparation of PU/GO Hybrid Wall Microcapsules and Their Self-Lubricating Properties for Epoxy Composites. Colloids Surf. A Physicochem. Eng. Asp..

[27] Asghar F., Shakoor B., Fatima S., Munir S., Razzaq H., Naheed S., Butler I. (2022). Fabrication and Prospective Applications of Graphene Oxide-Modified Nanocomposites for Wastewater Remediation. RSC Adv..

[28] Ramezanzadeh B., Niroumandrad S., Ahmadi A., Mahdavian M., Moghadam M.H.M. (2016). Enhancement of Barrier and Corrosion Protection Performance of an Epoxy Coating through Wet Transfer of Amino Functionalized Graphene Oxide. Corros. Sci..

[29] Ikram R., Jan B.M., Ahmad W. (2020). An Overview of Industrial Scalable Production of Graphene Oxide and Analytical Approaches for Synthesis and Characterization. J. Mater. Res. Technol..

[30] Fallahazad P. (2023). Rational and Key Strategies toward Enhancing the Performance of Graphene/Silicon Solar Cells. Mater. Adv..

[31] Gao Q., Liu S., Hou K., Li Z., Wang J. (2022). Graphene-Based Nanomaterials as Lubricant Additives: A Review. Lubricants.

[32] Fan S., Chen Y., Wu J., Xiao S., Chen G., Chu P.K. (2024). Structure, Superlubricity, Applications, and Chemical Vapor Deposition Methods of Graphene Solid Lubricants. Tribol. Int..

[33] Bian Y., Bian Z.-Y., Zhang J.-X., Ding A.-Z., Liu S.-L., Wang H. (2015). Effect of the Oxygen-Containing Functional Group of Graphene Oxide on the Aqueous Cadmium Ions Removal. Appl. Surf. Sci..

[34] Abdullah S.I., Ansari M.N.M. (2015). Mechanical Properties of Graphene Oxide (GO)/Epoxy Composites. HBRC J..

[35] Sun J., Du S. (2019). Application of Graphene Derivatives and Their Nanocomposites in Tribology and Lubrication: A Review. RSC Adv..

[36] Eksik O., Arvas M.B., Yavuz R. (2023). PAN-Based Nanofiber Reduced Graphene Oxide Electrodes for Supercapacitor Applications. J. Mater. Sci. Mater. Electron..

[37] Johari P., Shenoy V.B. (2011). Modulating Optical Properties of Graphene Oxide: Role of Prominent Functional Groups. ACS Nano.

[38] Liu L., Gao Y., Liu Q., Kuang J., Zhou D., Ju S., Han B., Zhang Z. (2013). High Mechanical Performance of Layered Graphene Oxide/Poly(Vinyl Alcohol) Nanocomposite Films. Small.

[39] Zhao H., Ding J., Yu H. (2018). Variation of Mechanical and Thermal Properties in Sustainable Graphene Oxide/Epoxy Composites. Sci. Rep..

[40] Pei S., Zhao J., Du J., Ren W., Cheng H.-M. (2010). Direct Reduction of Graphene Oxide Films into Highly Conductive and Flexible Graphene Films by Hydrohalic Acids. Carbon.

[41] Hu X., Yu Y., Hou W., Zhou J., Song L. (2013). Effects of Particle Size and pH Value on the Hydrophilicity of Graphene Oxide. Appl. Surf. Sci..

[42] Pacilé D., Meyer J.C., Fraile Rodríguez A., Papagno M., Gómez-Navarro C., Sundaram R.S., Burghard M., Kern K., Carbone C., Kaiser U. (2011). Electronic Properties and Atomic Structure of Graphene Oxide Membranes. Carbon.

[43] Cao C., Daly M., Singh C.V., Sun Y., Filleter T. (2015). High Strength Measurement of Monolayer Graphene Oxide. Carbon.

[44] Gómez-Navarro C., Meyer J.C., Sundaram R.S., Chuvilin A., Kurasch S., Burghard M., Kern K., Kaiser U. (2010). Atomic Structure of Reduced Graphene Oxide. Nano Lett..

[45] Francolini I., Perugini E., Silvestro I., Lopreiato M., Scotto d’Abusco A., Valentini F., Placidi E., Arciprete F., Martinelli A., Piozzi A. (2019). Graphene Oxide Oxygen Content Affects Physical and Biological Properties of Scaffolds Based on Chitosan/Graphene Oxide Conjugates. Materials.

[46] Ahmad H., Fan M., Hui D. (2018). Graphene Oxide Incorporated Functional Materials: A Review. Compos. Part B Eng..

[47] Tan Q., Fan Y., Song Z., Chen J., Chen L. (2022). Effects of Interlayer Spacing and Oxidation Degree of Graphene Oxide Nanosheets on Water Permeation: A Molecular Dynamics Study. J. Mol. Model..

[48] Su C., Loh K.P. (2013). Carbocatalysts: Graphene Oxide and Its Derivatives. Acc. Chem. Res..

[49] Yan H., Tao X., Yang Z., Li K., Yang H., Li A., Cheng R. (2014). Effects of the Oxidation Degree of Graphene Oxide on the Adsorption of Methylene Blue. J. Hazard. Mater..

[50] Li M., Liu C., Xie Y., Cao H., Zhao H., Zhang Y. (2014). The Evolution of Surface Charge on Graphene Oxide during the Reduction and Its Application in Electroanalysis. Carbon.

[51] Pareek S., Jain D., Shrivastava R., Dam S., Hussain S., Behera D. (2019). Tunable Degree of Oxidation in Graphene Oxide: Cost Effective Synthesis, Characterization and Process Optimization. Mater. Res. Express.

[52] Qi X., Pu K.-Y., Zhou X., Li H., Liu B., Boey F., Huang W., Zhang H. (2010). Conjugated-Polyelectrolyte-Functionalized Reduced Graphene Oxide with Excellent Solubility and Stability in Polar Solvents. Small.

[53] Tang L.-C., Wan Y.-J., Yan D., Pei Y.-B., Zhao L., Li Y.-B., Wu L.-B., Jiang J.-X., Lai G.-Q. (2013). The Effect of Graphene Dispersion on the Mechanical Properties of Graphene/Epoxy Composites. Carbon.

[54] Han D., Yan L., Chen W., Li W. (2011). Preparation of Chitosan/Graphene Oxide Composite Film with Enhanced Mechanical Strength in the Wet State. Carbohydr. Polym..

[55] Galpaya D., Wang M., George G., Motta N., Waclawik E., Yan C. (2014). Preparation of Graphene Oxide/Epoxy Nanocomposites with Significantly Improved Mechanical Properties. J. Appl. Phys..

[56] Wei X., Mao L., Soler-Crespo R.A., Paci J.T., Huang J., Nguyen S.T., Espinosa H.D. (2015). Plasticity and Ductility in Graphene Oxide through a Mechanochemically Induced Damage Tolerance Mechanism. Nat. Commun..

[57] Medhekar N.V., Ramasubramaniam A., Ruoff R.S., Shenoy V.B. (2010). Hydrogen Bond Networks in Graphene Oxide Composite Paper: Structure and Mechanical Properties. ACS Nano.

[58] Qiu Y., Guo F., Hurt R., Külaots I. (2014). Explosive Thermal Reduction of Graphene Oxide-Based Materials: Mechanism and Safety Implications. Carbon.

[59] Eigler S., Dimiev A.M. (2016). Functionalization and Reduction of Graphene Oxide. Graphene Oxide.

[60] Pei S., Cheng H.-M. (2012). The Reduction of Graphene Oxide. Carbon.

[61] Zhang P., Li Z., Zhang S., Shao G. (2018). Recent Advances in Effective Reduction of Graphene Oxide for Highly Improved Performance Toward Electrochemical Energy Storage. Energy Environ. Mater..

[62] Dolbin A.V., Khlistyuck M.V., Esel’son V.B., Gavrilko V.G., Vinnikov N.A., Basnukaeva R.M., Maluenda I., Maser W.K., Benito A.M. (2016). The Effect of the Thermal Reduction Temperature on the Structure and Sorption Capacity of Reduced Graphene Oxide Materials. Appl. Surf. Sci..

[63] Sun W., Wang L., Yang Z., Zhu T., Wu T., Dong C., Liu G. (2018). Tuning the Oxidation Degree of Graphite toward Highly Thermally Conductive Graphite/Epoxy Composites. Chem. Mater..

[64] Shudo Y., Karim M.R., Ohtani R., Nakamura M., Hayami S. (2018). Hybrids from the Π−π Stacking of Graphene Oxide and Aromatic Sulfonic Compounds for Improved Proton Conductivity. ChemElectroChem.

[65] Wei N., Lv C., Xu Z. (2014). Wetting of Graphene Oxide: A Molecular Dynamics Study. Langmuir.

[66] Fu X.-K., Cao H.-B., An Y.-L., Zhou H.-D., Shi Y.-P., Hou G.-L., Ha W. (2022). Bioinspired Hydroxyapatite Coating Infiltrated with a Graphene Oxide Hybrid Supramolecular Hydrogel Orchestrates Antibacterial and Self-Lubricating Performance. ACS Appl. Mater. Interfaces.

[67] Dasari B.L., Morshed M., Nouri J.M., Brabazon D., Naher S. (2018). Mechanical Properties of Graphene Oxide Reinforced Aluminium Matrix Composites. Compos. Part B Eng..

[68] Gupta B., Kumar N., Panda K., Dash S., Tyagi A.K. (2016). Energy Efficient Reduced Graphene Oxide Additives: Mechanism of Effective Lubrication and Antiwear Properties. Sci. Rep..

[69] Jena G., Philip J. (2022). A Review on Recent Advances in Graphene Oxide-Based Composite Coatings for Anticorrosion Applications. Prog. Org. Coat..

[70] Chen H., Ba Z., Qiao D., Feng D., Song Z., Zhang J. (2020). Study on the Tribological Properties of Graphene Oxide Composite Films by Self-Assembly. Tribol. Int..

[71] Ma L., Xie G., Luo P., Zhang L., Fan Y., He Y. (2022). Dispersion Stability of Graphene Oxide in Extreme Environments and Its Applications in Shale Exploitation. ACS Sustain. Chem. Eng..

[72] Dong H.S., Qi S.J. (2015). Realising the Potential of Graphene-Based Materials for Biosurfaces—A Future Perspective. Biosurface and Biotribol..

[73] Miao C., Tang J., Yang K., Xiao N., Shao Z., Zhang F., Zhang H., Xiong Y., Xiong B., Chen H. (2023). Recent Progress on the Tribological Applications of Solid Lubricants. J. Tribol..

[74] Yang X., Wang Q., Zhu K., Ye K., Wang G., Cao D., Yan J. (2021). 3D Porous Oxidation-Resistant MXene/Graphene Architectures Induced by In Situ Zinc Template toward High-Performance Supercapacitors. Adv. Funct. Mater..

[75] Ayyagari A.V., Mutyala K.C., Sumant A.V. (2020). Towards Developing Robust Solid Lubricant Operable in Multifarious Environments. Sci. Rep..

[76] Berman D., Erdemir A., Sumant A.V. (2014). Graphene: A New Emerging Lubricant. Mater. Today.

[77] Alazemi A.A., Dysart A.D., Shaffer S.J., Pol V.G., Stacke L.-E., Sadeghi F. (2017). Novel Tertiary Dry Solid Lubricant on Steel Surfaces Reduces Significant Friction and Wear under High Load Conditions. Carbon.

[78] Chen L., Wu G., Huang Y., Bai C., Yu Y., Zhang J. (2021). High Loading Capacity and Wear Resistance of Graphene Oxide/Organic Molecule Assembled Multilayer Film. Front. Chem..

[79] Dhanola A., Gajrani K.K. (2023). Novel Insights into Graphene-Based Sustainable Liquid Lubricant Additives: A Comprehensive Review. J. Mol. Liq..

[80] Liu Y., Chen X., Li J., Luo J. (2019). Enhancement of Friction Performance Enabled by a Synergetic Effect between Graphene Oxide and Molybdenum Disulfide. Carbon.

[81] Wu W., Liu J., Li Z., Zhao X., Liu G., Liu S., Ma S., Li W., Liu W. (2021). Surface-Functionalized nanoMOFs in Oil for Friction and Wear Reduction and Antioxidation. Chem. Eng. J..

[82] Farsadi M., Bagheri S., Ismail N.A. (2017). Nanocomposite of Functionalized Graphene and Molybdenum Disulfide as Friction Modifier Additive for Lubricant. J. Mol. Liq..

[83] Li X., Lu H., Guo J., Tong Z., Dong G. (2018). Synergistic Water Lubrication Effect of Self-Assembled Nanofilm and Graphene Oxide Additive. Appl. Surf. Sci..

[84] Zhao J., Li Y., Wang Y., Mao J., He Y., Luo J. (2017). Mild Thermal Reduction of Graphene Oxide as a Lubrication Additive for Friction and Wear Reduction. RSC Adv..

[85] Xie H., Jiang B., Dai J., Peng C., Li C., Li Q., Pan F. (2018). Tribological Behaviors of Graphene and Graphene Oxide as Water-Based Lubricant Additives for Magnesium Alloy/Steel Contacts. Materials.

[86] Kaleli H., Demirtaş S., Uysal V., Karnis I., Stylianakis M.M., Anastasiadis S.H., Kim D.-E. (2021). Tribological Performance Investigation of a Commercial Engine Oil Incorporating Reduced Graphene Oxide as Additive. Nanomaterials.

[87] Khatai S., Sahoo A.K., Kumar R., Panda A. (2024). Performance assessment of graphene oxide based nano-cutting fluids during sustainable hard turning through synthesis, characterization and machinability investigation. Diam. Relat. Mater..

[88] Zeng Q., Zhang W. (2023). A Systematic Review of the Recent Advances in Superlubricity Research. Coatings.

[89] Xing Z., Zhang J., Kaindl R., Zhang B. (2025). Solid superlubricity of diamond-like carbon films: A review. Surf. Sci. Technol..

[90] Yi S., Li N., Solanki S., Mo J., Ding S. (2019). Effects of graphene oxide nanofluids on cutting temperature and force in machining Ti-6Al-4V. Int. J. Adv. Manuf. Technol..

[91] Goralka C., Bridges J., Jahan M., Sidebottom M., Cameron T., Lu Y., Ye Z. (2022). Friction and Wear Reduction of Tungsten Carbide and Titanium Alloy Contacts via Graphene Nanolubricant. Lubricants.

[92] Sarno M., Scarpa D., Senatore A., Ahmed Abdalglil Mustafa W. (2020). rGO/GO Nanosheets in Tribology: From the State of the Art to the Future Prospective. Lubricants.

[93] Yoo B.M., Shin H.J., Yoon H.W., Park H.B. (2014). Graphene and Graphene Oxide and Their Uses in Barrier Polymers. J. Appl. Polym. Sci..

[94] Jena G., Anandkumar B., Sofia S., George R.P., Philip J. (2020). Fabrication of Silanized GO Hybrid Coating on 316L SS with Enhanced Corrosion Resistance and Antibacterial Properties for Marine Applications. Surf. Coat. Technol..

[95] Hu Q., Li X., Zhao G., Ruan Y., Wang G., Ding Q. (2023). Effects of Graphene Oxide on Tribological Properties of Micro-Arc Oxidation Coatings on Ti-6Al-4V. Coatings.

[96] Mo M., Zhao W., Chen Z., Yu Q., Zeng Z., Wu X., Xue Q. (2015). Excellent Tribological and Anti-Corrosion Performance of Polyurethane Composite Coatings Reinforced with Functionalized Graphene and Graphene Oxide Nanosheets. RSC Adv..

[97] Ramkumar N.P., Sharma S.C., Adarsha H., Keshavamurthy R. (2025). Tribological Performance of PEEK/GO Nanocomposites Fabricated via Stereolithography. J. Bio-Tribo-Corros..

[98] Chen X., Meng D., Wang B., Li B.-W., Li W., Bielawski C.W., Ruoff R.S. (2016). Rapid Thermal Decomposition of Confined Graphene Oxide Films in Air. Carbon.

[99] Gong X., Liu G., Li Y., Yu D.Y.W., Teoh W.Y. (2016). Functionalized-Graphene Composites: Fabrication and Applications in Sustainable Energy and Environment. Chem. Mater..

[100] Abusultan A., Abunahla H., Halawani Y., Mohammad B., Alamoodi N., Alazzam A. (2022). Artificial Intelligence-Aided Low Cost and Flexible Graphene Oxide-Based Paper Sensor for Ultraviolet and Sunlight Monitoring. Nanoscale Res. Lett..

[101] Nemati N., Emamy M., Yau S., Kim J.-K., Kim D.-E. (2016). High Temperature Friction and Wear Properties of Graphene Oxide/Polytetrafluoroethylene Composite Coatings Deposited on Stainless Steel. RSC Adv..

[102] Shah R., Kausar A., Muhammad B., Shah S. (2015). Progression from Graphene and Graphene Oxide to High Performance Polymer-Based Nanocomposite: A Review. Polym.-Plast. Technol. Eng..

[103] Wang C., Sun J., He J., Ge C. (2022). Friction-Induced Motion Evolution of Reduced Graphene Oxide-Al2O3 at Contact Interface to Achieve Superior Lubrication Performance. Appl. Surf. Sci..

[104] Zhang M., Yu Y., Li L., Zhou H., Gong L., Zhou H. (2024). A Molecular Dynamics Assisted Insight on Damping Enhancement in Carbon Fiber Reinforced Polymer Composites with Oriented Multilayer Graphene Oxide Coatings. Microstructures.

[105] Wang Y., Meng Z. (2021). Mechanical and Viscoelastic Properties of Wrinkled Graphene Reinforced Polymer Nanocomposites—Effect of Interlayer Sliding within Graphene Sheets. Carbon.

[106] Tong L.B., Zhang J.B., Xu C., Wang X., Song S.Y., Jiang Z.H., Kamado S., Cheng L.R., Zhang H.J. (2016). Enhanced Corrosion and Wear Resistances by Graphene Oxide Coating on the Surface of Mg-Zn-Ca Alloy. Carbon.

[107] Miao X., Liu S., Ma L., Yang Y., Zhu J., Li Z., Wang J. (2022). Ti_3_C_2_-Graphene Oxide Nanocomposite Films for Lubrication and Wear Resistance. Tribol. Int..

[108] Voevodin A.A., Muratore C., Aouadi S.M. (2014). Hard Coatings with High Temperature Adaptive Lubrication and Contact Thermal Management: Review. Surf. Coat. Technol..

[109] Chen Z., Wei P., Zhang S., Lu B., Zhang L., Yang X., Huang K., Huang Y., Li X., Zhao Q. (2020). Graphene reinforced nickel-based superalloy composites fabricated by additive manufacturing. Mater. Sci. Eng. A.

[110] Yan S., Zhai W., Xiao J., Zhai W. (2022). Ahmed Mohamed Mahmoud Ibrahim, Graphene oxide decorated spherical powder for Ni superalloy with high yield strength and ductility. Mater. Sci. Eng. A.

[111] Porwal H., Grasso S., Reece M.J. (2013). Review of Graphene–Ceramic Matrix Composites. Adv. Appl. Ceram..

[112] Xu Y., Zhou P., Chen Q., Liu Z., Wang X., Deng M., Zhou H., Han Y., Yao P. (2024). The Effect of Copper Particles Coated with Graphene Oxide on Tribological Properties and Tribo-Layers of Copper Metal Matrix Composites. Tribol. Int..

[113] Sarath P.S., Reghunath R., Thomas S., Haponiuk J.T., George S.C., George S.C., Haponiuk J.T., Thomas S., Reghunath R., Sarath P.S. (2023). 8—Tribology of Graphene-Based Polymeric Systems. Tribology of Polymers, Polymer Composites, and Polymer Nanocomposites.

[114] Kumar H.G.P., Xavior M.A. (2014). Graphene Reinforced Metal Matrix Composite (GRMMC): A Review. Procedia Eng..

[115] Markandan K., Chin J.K., Tan M.T.T. (2017). Recent Progress in Graphene Based Ceramic Composites: A Review. J. Mater. Res..

[116] Xu H., Liu Y., Wang K. (2024). Preparation high-performance SiC ceramic reinforced with 3D hybrid graphene oxide-carbon nanotube by direct ink writing and liquid silicon infiltration. J. Eur. Ceram. Soc..

[117] Pathak A.K., Borah M., Gupta A., Yokozeki T., Dhakate S.R. (2016). Improved Mechanical Properties of Carbon Fiber/Graphene Oxide-Epoxy Hybrid Composites. Compos. Sci. Technol..

[118] Li H., Li T., Zhang T., Zhu J., Deng W., He D. (2022). Construction and Adsorption Performance Study of GO-CNT/Activated Carbon Composites for High Efficient Adsorption of Pollutants in Wastewater. Polymers.

[119] Kim J., Kim J. (2023). PBO Fiber Grafted rGO Aerogel/BN/PBO Composites with Highly Improved Electromagnetic Interference Shielding Effectiveness and through-Plane Thermal Conductivity. Polym. Test..

[120] Wang Y., Jiang D., Ma X., Zhang Y., Fu P., Du F. (2024). Exfoliated MoS2 Anchored on Graphene Oxide Nanosheets for Enhancing Thermoelectric Properties of Single-Walled Carbon Nanotubes. Ceram. Int..

[121] Singh S., Rathi K., Pal K. (2018). Synthesis, Characterization of Graphene Oxide Wrapped Silicon Carbide for Excellent Mechanical and Damping Performance for Aerospace Application. J. Alloys Compd..

[122] Zhang C., Nieto A., Agarwal A. (2016). Ultrathin Graphene Tribofilm Formation during Wear of Al_2_O_3_–Graphene Composites. Nanomater. Energy.

[123] Nassef M.G.A., Soliman M., Nassef B.G., Daha M.A., Nassef G.A. (2022). Impact of Graphene Nano-Additives to Lithium Grease on the Dynamic and Tribological Behavior of Rolling Bearings. Lubricants.

[124] Wang Q., Ramírez C., Watts C.S., Borrero-López O., Ortiz A.L., Sheldon B.W., Padture N.P. (2020). Fracture, Fatigue, and Sliding-Wear Behavior of Nanocomposites of Alumina and Reduced Graphene-Oxide. Acta Mater..

[125] Zhao P., Yan J., Yan H., Zhou S., Huang J., Wu X., Zhao G., Liu Y. (2023). Wear and Corrosion Resistance of Self-Healing Epoxy Coatings Filled by Polydopamine-Modified Graphene Oxide Assembly of Polysulfone Double-Walled Microcapsules. Prog. Org. Coat..

[126] Sánchez-López L., Ropero de Torres N., Chico B., Soledad Fagali N., de los Ríos V., Escudero M.L., García-Alonso M.C., Lozano R.M. (2023). Effect of Wear-Corrosion of Reduced Graphene Oxide Functionalized with Hyaluronic Acid on Inflammatory and Proteomic Response of J774A.1 Macrophages. Metals.

[127] Daly M., Cao C., Sun H., Sun Y., Filleter T., Singh C.V. (2016). Interfacial Shear Strength of Multilayer Graphene Oxide Films. ACS Nano.

[128] Chen D., Feng H., Li J. (2012). Graphene Oxide: Preparation, Functionalization, and Electrochemical Applications. Chem. Rev..

[129] Zhang L., Hou G., Zhai W., Ai Q., Feng J., Zhang L., Si P., Ci L. (2018). Aluminum/Graphene Composites with Enhanced Heat-Dissipation Properties by in-Situ Reduction of Graphene Oxide on Aluminum Particles. J. Alloys Compd..

[130] Wang M., Li Z., Wang J., Yang S. (2023). Iron Ions Induced Self-Assembly of Graphene Oxide Lubricating Coating with Self-Adapting Low Friction Characteristics. Carbon.

[131] Zhang L., Shi Q., Ge X. (2024). Comparative Study of the Friction Behavior of Functionalized Graphene Oxide Additives Under Electric Stimulations. Lubricants.

[132] Cao N., Zhang Y. (2015). Study of Reduced Graphene Oxide Preparation by Hummers’ Method and Related Characterization. J. Nanomater..

[133] Zaaba N.I., Foo K.L., Hashim U., Tan S.J., Liu W.-W., Voon C.H. (2017). Synthesis of Graphene Oxide Using Modified Hummers Method: Solvent Influence. Procedia Eng..

[134] Kotsyubynsky V.O., Boychuk V.M., Budzulyak I.M., Rachiy B.I., Hodlevska M.A., Kachmar A.I., Hodlevsky M.A. (2021). Graphene Oxide Synthesis Using Modified Tour Method. Adv. Nat. Sci. Nanosci. Nanotechnol..

[135] Zhu P., Shen M., Xiao S., Zhang D. (2011). Experimental Study on the Reducibility of Graphene Oxide by Hydrazine Hydrate. Phys. B Condens. Matter.

[136] De Silva K.K.H., Huang H.-H., Yoshimura M. (2018). Progress of Reduction of Graphene Oxide by Ascorbic Acid. Appl. Surf. Sci..

[137] Thomas H.R., Phillips D.J., Wilson N.R., Gibson M.I., Rourke J.P. (2015). One-Step Grafting of Polymers to Graphene Oxide. Polym. Chem..

[138] Cai Y., Fadil Y., Jasinski F., Thickett S.C., Agarwal V., Zetterlund P.B. (2019). Miniemulsion Polymerization Using Graphene Oxide as Surfactant: In Situ Grafting of Polymers. Carbon.

[139] Yi M., Shen Z. (2015). A Review on Mechanical Exfoliation for the Scalable Production of Graphene. J. Mater. Chem. A.

[140] Qi X., Zhou T., Deng S., Zong G., Yao X., Fu Q. (2014). Size-Specified Graphene Oxide Sheets: Ultrasonication Assisted Preparation and Characterization. J. Mater. Sci..

[141] Casallas Caicedo F.M., Vera López E., Agarwal A., Drozd V., Durygin A., Franco Hernandez A., Wang C. (2020). Synthesis of Graphene Oxide from Graphite by Ball Milling. Diam. Relat. Mater..

[142] Tas M., Altin Y., Celik Bedeloglu A. (2019). Reduction of Graphene Oxide Thin Films Using a Stepwise Thermal Annealing Assisted by L-Ascorbic Acid. Diam. Relat. Mater..

[143] El-Hossary F.M., Ghitas A., El-Rahman A.M.A., Shahat M.A., Fawey M.H. (2021). The Effective Reduction of Graphene Oxide Films Using RF Oxygen Plasma Treatment. Vacuum.

[144] Qahtan T.F., Owolabi T.O., Alhakami F.S., Saleh T.A. (2025). Low-Energy Argon Ion Beam Irradiation for the Surface Modification and Reduction of Graphene Oxide: Insights from XPS. Radiat. Phys. Chem..

[145] Lowe S.E., Zhong Y.L. (2016). Challenges of Industrial-Scale Graphene Oxide Production. Graphene Oxide.

[146] Marchesini S., Paton K.R., Pollard A.J. (2024). Navigating the Frontiers of Graphene Quality Control to Enable Product Optimisation and Market Confidence. Nano Futures.

[147] Thakur K., Kandasubramanian B. (2019). Graphene and Graphene Oxide-Based Composites for Removal of Organic Pollutants: A Review. J. Chem. Eng. Data.

[148] Pendolino F., Armata N., Pendolino F., Armata N. (2017). Regulation and Environmental Aspects of Graphene Oxide. Graphene Oxide in Environmental Remediation Process.

[149] Donato K.Z., Tan H.L., Marangoni V.S., Martins M.V.S., Ng P.R., Costa M.C.F., Jain P., Lee S.J., Koon G.K.W., Donato R.K. (2023). Graphene Oxide Classification and Standardization. Sci. Rep..

[150] Barroso-Bujans F., Alegría A., Pomposo J.A., Colmenero J. (2013). Thermal Stability of Polymers Confined in Graphite Oxide. Macromolecules.

[151] Khan F., Khan M.S., Kamal S., Arshad M., Ahmad S.I., Nami S.A.A. (2020). Recent Advances in Graphene Oxide and Reduced Graphene Oxide Based Nanocomposites for the Photodegradation of Dyes. J. Mater. Chem. C.

[152] Shams M., Guiney L.M., Huang L., Ramesh M., Yang X., Hersam M.C., Chowdhury I. (2019). Influence of Functional Groups on the Degradation of Graphene Oxide Nanomaterials. Environ. Sci. Nano.

[153] Choi Y.R., Yoon Y.-G., Choi K.S., Kang J.H., Shim Y.-S., Kim Y.H., Chang H.J., Lee J.-H., Park C.R., Kim S.Y. (2015). Role of Oxygen Functional Groups in Graphene Oxide for Reversible Room-Temperature NO2 Sensing. Carbon.

[154] Guardia L., Villar-Rodil S., Paredes J.I., Rozada R., Martínez-Alonso A., Tascón J.M.D. (2012). UV Light Exposure of Aqueous Graphene Oxide Suspensions to Promote Their Direct Reduction, Formation of Graphene–Metal Nanoparticle Hybrids and Dye Degradation. Carbon.

[155] Hua Z., Tang Z., Bai X., Zhang J., Yu L., Cheng H. (2015). Aggregation and Resuspension of Graphene Oxide in Siulated Natural Surface Aquatic Environments. Environ. Pollut..

[156] Bolibok P., Wiśniewski M., Roszek K., Terzyk A.P. (2017). Controlling Enzymatic Activity by Immobilization on Graphene Oxide. Sci. Nat..

[157] Losic D., Farivar F., Yap P.L., Tung T.T., Nine M.J. (2022). New Insights on Energetic Properties of Graphene Oxide (GO) Materials and Their Safety and Environmental Risks. Sci. Total Environ..

[158] Vimalanathan K., Scott J., Pan X., Luo X., Rahpeima S., Sun Q., Zou J., Bansal N., Prabawati E., Zhang W. (2022). Continuous Flow Fabrication of Green Graphene Oxide in Aqueous Hydrogen Peroxide. Nanoscale Adv..

[159] Shao J.-J., Lv W., Yang Q.-H. (2014). Self-Assembly of Graphene Oxide at Interfaces. Adv. Mater..

[160] Li X., Liu Y.M., Li W.G., Li C.Y., Sanjayan J.G., Duan W.H., Li Z. (2017). Effects of Graphene Oxide Agglomerates on Workability, Hydration, Microstructure and Compressive Strength of Cement Paste. Constr. Build. Mater..

[161] Yang Y., Cao J., Wu P., Luo T., Liang T., Yin H., Yuan K. (2024). Effect of Temperature on Interface Debonding Behavior of Graphene/Graphene-Oxide on Cement-Based Composites. Surf. Interfaces.

[162] Ye M., Gao J., Xiao Y., Xu T., Zhao Y., Qu L. (2017). Metal/Graphene Oxide Batteries. Carbon.

[163] Panda S., Rout T.K., Prusty A.D., Ajayan P.M., Nayak S. (2018). Electron Transfer Directed Antibacterial Properties of Graphene Oxide on Metals. Adv. Mater..

